# Proteomic analysis of X-linked dystonia parkinsonism disease striatal neurons reveals altered RNA metabolism and splicing

**DOI:** 10.1016/j.nbd.2023.106367

**Published:** 2023-11-30

**Authors:** Kizito-Tshitoko Tshilenge, Joanna Bons, Carlos Galicia Aguirre, Cristian Geronimo-Olvera, Samah Shah, Jacob Rose, Akos A. Gerencser, Sally K. Mak, Michelle E. Ehrlich, D. Cristopher Bragg, Birgit Schilling, Lisa M. Ellerby

**Affiliations:** a The Buck Institute for Research on Aging, Novato, California 94945, USA; b University of Southern California, Leonard Davis School of Gerontology, 3715 McClintock Ave, Los Angeles, CA 90893, USA; c Department of Neurology, Icahn School of Medicine at Mount Sinai, New York, NY 10029, USA; d Department of Neurology, Massachusetts General Hospital and Harvard Medical School, Boston, MA, USA; e Department of Neurology, The Collaborative Center for X-linked Dystonia-Parkinsonism, Massachusetts General Hospital, Charlestown, MA, USA.

**Keywords:** Neurodegeneration, X-linked dystonia parkinsonism disease, Induced pluripotent stem cells, Medium spiny neurons, Quantitative proteomics, RNA metabolism, RNA splicing

## Abstract

X-linked dystonia-parkinsonism (XDP) is a rare neurodegenerative disease endemic to the Philippines. The genetic cause for XDP is an insertion of a SINE-VNTR-Alu (SVA)-type retrotransposon within intron 32 of TATA-binding protein associated factor 1 (TAF1) that causes an alteration of TAF1 splicing, partial intron retention, and decreased transcription. Although TAF1 is expressed in all organs, medium spiny neurons (MSNs) within the striatum are one of the cell types most affected in XDP. To define how mutations in the *TAF1* gene lead to MSN vulnerability, we carried out a proteomic analysis of human XDP patient-derived neural stem cells (NSCs) and MSNs derived from induced pluripotent stem cells. NSCs and MSNs were grown in parallel and subjected to quantitative proteomic analysis in data-independent acquisition mode on the Orbitrap Eclipse Tribrid mass spectrometer. Subsequent functional enrichment analysis demonstrated that neurodegenerative disease-related pathways, such as Huntington’s disease, spinocerebellar ataxia, cellular senescence, mitochondrial function and RNA binding metabolism, were highly represented. We used weighted coexpression network analysis (WGCNA) of the NSC and MSN proteomic data set to uncover disease-driving network modules. Three of the modules significantly correlated with XDP genotype when compared to the non-affected control and were enriched for DNA helicase and nuclear chromatin assembly, mitochondrial disassembly, RNA location and mRNA processing. Consistent with aberrant mRNA processing, we found splicing and intron retention of TAF1 intron 32 in XDP MSN. We also identified TAF1 as one of the top enriched transcription factors, along with YY1, ATF2, USF1 and MYC. Notably, YY1 has been implicated in genetic forms of dystonia. Overall, our proteomic data set constitutes a valuable resource to understand mechanisms relevant to TAF1 dysregulation and to identify new therapeutic targets for XDP.

## 1. Introduction

X-linked dystonia-parkinsonism (XDP) is a devastating hereditary neurodegenerative disease endemic to the island of Panay, Philippines (Lee et al., 2002; Lee et al., 2011). XDP affects males with an estimated prevalence of 5.74 cases per 100,000 individuals in Panay, and cases of symptomatic female XDP carriers have been reported (Domingo et al., 2015; Lee et al., 2011). The first clinical signs of XDP usually manifest in the third or fourth decade of life, with a movement disorder characterized by dystonic symptoms that co-exist or are replaced by a parkinsonism phenotype beyond the 10th year of the disease (Lee et al., 1991; Lee et al., 2002; Lee et al., 2011). A hallmark of XDP neuropathology is the sequential degeneration of medium spiny neurons (MSNs) within the striatum (Goto et al., 2013; Goto et al., 2005; Waters et al., 1993), perhaps first in the striosomal compartment, with some similarities to the pathology of Huntington’s disease (HD) (Vonsattel et al., 1985). Other regions of the brain may also be involved, such as cortex, substantia nigra and cerebellum (Arasaratnam et al., 2021; Bruggemann et al., 2016; Petrozziello et al., 2020b). There are no effective treatments to delay the onset or slow the progression of XDP.

Previous genetic studies mapped the XDP locus to a region on the X-chromosome that was recently narrowed to a 219.7 kb segment in which *TAF1* is the only gene (Makino et al., 2007; Nemeth et al., 1999; Nolte et al., 2003). All probands characterized to date appear to share a 13-marker haplotype clustered in noncoding regions within and around *TAF1*, which encodes the TATA-binding protein associated factor-1 (TAF1) protein (Aneichyk et al., 2018). Although, it is difficult to demonstrate the functional consequences of these variants in the disease, a ~ 2.6-kb SINE-VNTR-Alu-CCCTCT (SVA)-type retrotransposon (Hancks and Kazazian Jr., 2010) in intron 32 of *TAF1* (Makino et al., 2007) has a variable number of hexameric repeats (Westenberger et al., 2019) among XDP patients. An increasing repeat number of the SVA is strongly correlated with earlier age at disease onset (Bragg et al., 2017). Like other repeat expansion disorders, XDP CCCTCT repeat is subject to somatic expansions (Campion et al., 2022).

Human patient-derived induced pluripotent stem cells (iPSCs) have been used to model XDP (Al Ali et al., 2021; Aneichyk et al., 2018; Bragg et al., 2017; Capetian et al., 2018; Ito et al., 2016; Petrozziello et al., 2020a; Rakovic et al., 2018; Vaine et al., 2017) and can recapitulate features of XDP pathology. For example, transcriptional profiling of iPSC neural derivatives has revealed that XDP cells exhibit aberrant *TAF1* splicing, increased partial retention of intron 32, and decreased expression of full-length *TAF1* mRNA, all of which can be rescued by CRISPR/Cas-based excision of the SVA (Aneichyk et al., 2018; Ito et al., 2016). These observations may account for the lower levels of TAF1 expression documented across post-mortem brain tissue and cell models, thereby suggesting that XDP may in part be due to the loss of function of TAF1 (Al Ali et al., 2021; Aneichyk et al., 2018; Domingo et al., 2016; Makino et al., 2007; Rakovic et al., 2018).

**Fig. 1.**
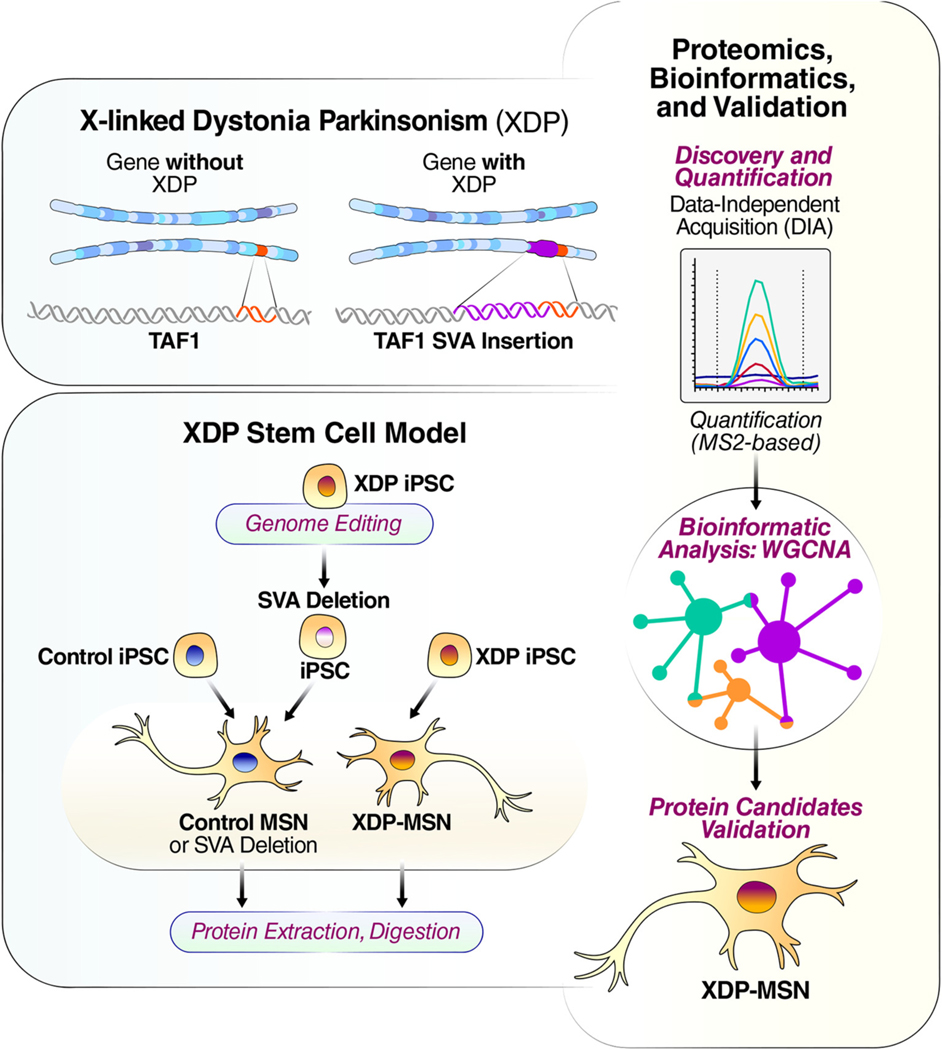
Schematic representation of XDP proteomics workflow. XDP is a neurodegenerative disease caused by a 13-marker haplotype clustered in and around *TAF1* that leads to alteration of TAF1 expression. Insertion of a SINE-VNTR-Alu (SVA)-type retrotransposon within an intron of *TAF1* is linked to a dysregulation of TAF1 splicing, partial intron retention, and transcriptional interference. The striatum is affected in XDP. iPSCs-derived from XDP patients were differentiated into NSCs and MSNs from XDP patients, matched relative controls (healthy sons) and isogenic lines in which the SVA was deleted. Comprehensive quantitative proteomic analysis with deep coverage using a combination of data dependent acquisition (DDA, for comprehensive spectral library building) and DIA (for accurate quantification) on the Orbitrap Eclipse Tribrid mass spectrometer. After protein identification and quantification processing of the DIA data, bioinformatic analyses, including WGCNA, were used to identify molecular pathways and networks relevant to XDP.

TAF1 encodes a transcription factor, TATA-binding protein–associated factor-1, a subunit of the transcription factor II D (TFIID) complex involved in transcription initiation by RNA polymerase II (Malkowska et al., 2013). Like many triplet-repeat diseases, XDP pathogenesis is likely driven by a number of pathological mechanisms (Ellerby, 2019). One approach to gain insights into how TAF1 mutations cause XDP is to define the proteome of patient-derived iPSCs and, particularly, to model the relevant neuronal cell types impacted in XDP. To date, the XDP proteome of the vulnerable striatal MSN has not been defined. Recently, our group showed the value of integrating a proteomics data set from iPSC-derived HD MSNs to identify novel mechanisms and therapeutic targets in dysregulated human HD MSNs (Tshilenge et al., 2023). In the current study, XDP patient-derived iPSC were differentiated into NSCs and MSNs from XDP patients, matched controls and isogenic lines subjected to CRISPR/Cas9-based excision of the SVA (Aneichyk et al., 2018). We performed comprehensive quantitative proteomics (Bruderer et al., 2017; Collins et al., 2017; Gillet et al., 2012) to define the proteomic changes within XDP NSCs and MSNs (Fig. 1).

## 2. Results

### 2.1. Generation and characterization of XDP-iPSC-derived NSCs and MSNs

To investigate the molecular mechanisms underlying XDP neuropathogenesis, we used established male iPSC lines from XDP patients and unaffected relatives (Aneichyk et al., 2018; Ito et al., 2016) (Table 1). To encompass the heterogeneity of the disease with respect to rates of progression and hexameric repeats, we used XDP iPSCs derived from two families, with each containing the carrier XDP-14i-Family 1 (33363.C, hexameric repeat length 40) and XDP-4i-Family 2 (33109.2B, hexameric repeat length 36) and the respective controls Ctrl-13i-Family 1 (33362.D) and Ctrl-8i-Family 2 (33114.B). In addition, we included an XDP isogenic line in which CRISPR/Cas9 editing deleted the SVA (SVA-edited-1A4i-Family 2, 33109.2G) (Aneichyk et al., 2018) (Table 1, Supplementary Fig. 1).

**Fig. 2.**
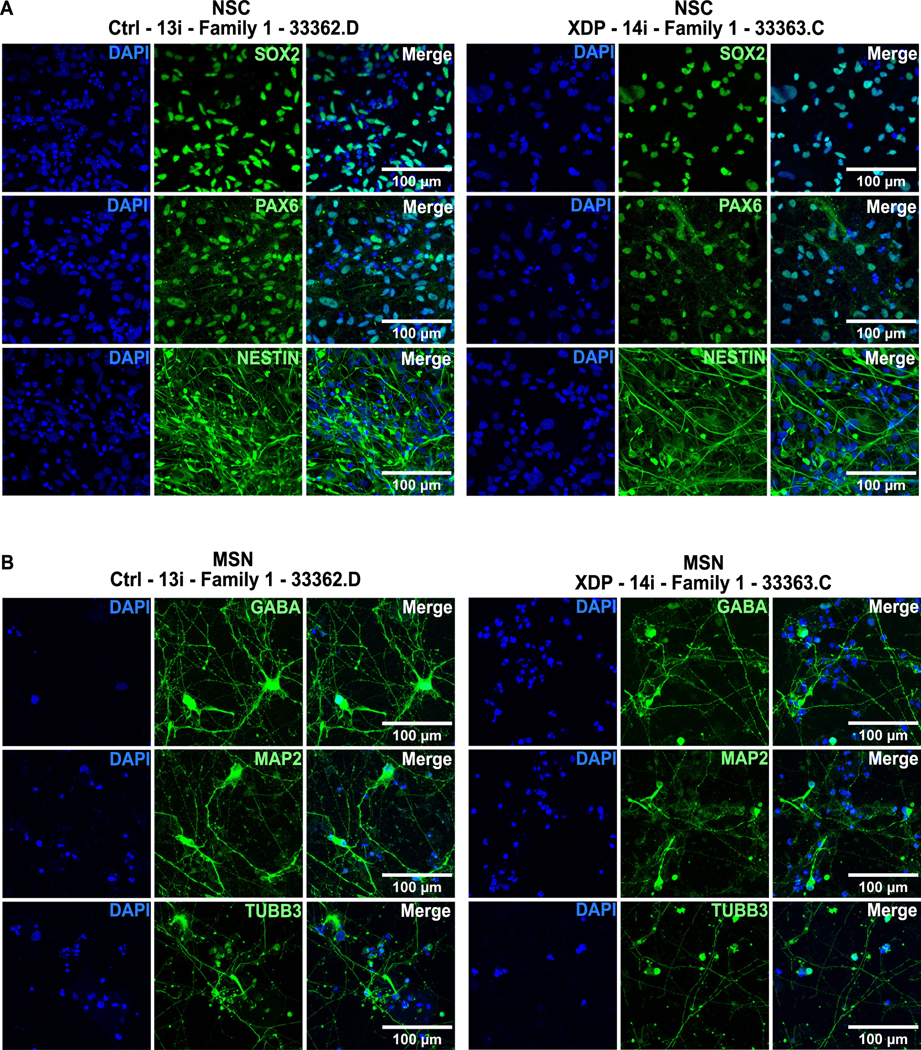
Generation and characterization of XDP-derived NSCs and MSNs. *A*, NSCs derived from XDP patients and matched controls were immunostained with SOX2, PAX6, and NESTIN. *B*, MSNs derived from XDP patients and matched controls were immunostained with GABA, MAP2, and TUBB3.

Given that XDP neurodegeneration affects striatal tissue with loss of GABAergic inhibitory neurons, MSNs, we used established protocols to differentiate XDP iPSC into NSCs and MSNs. The differentiation process was carried out in the presence of Activin A to induce dorsoventral prepatterning toward a lateral ganglionic eminence (LGE) identity (Arber et al., 2015; Kemp et al., 2016; Telezhkin et al., 2016). The NSC identity was confirmed by measuring the expression of SOX2, PAX6, and NESTIN with immunofluorescence (Fig. 2A). After neural differentiation of the control, XDP and SVA-edited NSCs, we observed the expression of mature neuronal markers, including GABA, MAP-2, and TUBB3 (Fig. 2B). Further, these cultures are DARPP-32 positive (Supplementary Fig. 2), have MSN markers as described previously (Tshilenge et al., 2023) and the cultures are similar to developing human MSNs (Galicia Aguirre, et al., 2023).

### 2.2. Analysis of XDP-associated proteome alteration by quantitative proteomics

To gain insight into XDP biology and identify disease-associated proteome alterations, we conducted a comprehensive label-free quantitative proteomics analysis of NSCs and MSNs by LC-MS/MS in DIA mode (Bruderer et al., 2017; Collins et al., 2017; Gillet et al., 2012) on the Orbitrap Eclipse Tribrid mass spectrometer. Neural cells were grown in parallel with five replicates for each genotype (fathers with XDP, healthy sons, SVA-corrected), making a total of 50 samples, with *N* = 25 for NSCs and N = 25 for MSNs (Table 1). After extraction and digestion of the intracellular proteins, DIA measurements were performed, and information collected in MS/MS spectra was used to accurately quantify the proteins with very high reproducibility. DIA data were processed using Spectronaut (Biognosys) with sample-specific hybrid DDA-DIA spectral libraries (Supplementary Fig. 3 A; Supplementary Table S2, S3) to achieve high quantification performances (Supplementary Fig. 3B-E). Indeed, the efficient retention time regression in Spectronaut calibration enabled improvement of the specificity of the DIA assays, and protein quantification was highly reproducible as illustrated by the proportion of proteins with a coefficient of variation below 20% for ~50–80% of all protein groups depending on genotype/patient groups (Supplementary Fig. 3B-E). Overall, we identified and quantified 5052 and 4113 unique protein groups (≥ 2 unique peptides per protein, FDR ≤ 0.01) for NSCs (Supplementary Table S4) and MSNs (Supplementary Table S5), respectively, and thus providing comprehensive and deep coverage of the NSC and MSN proteomes.

**Fig. 3.**
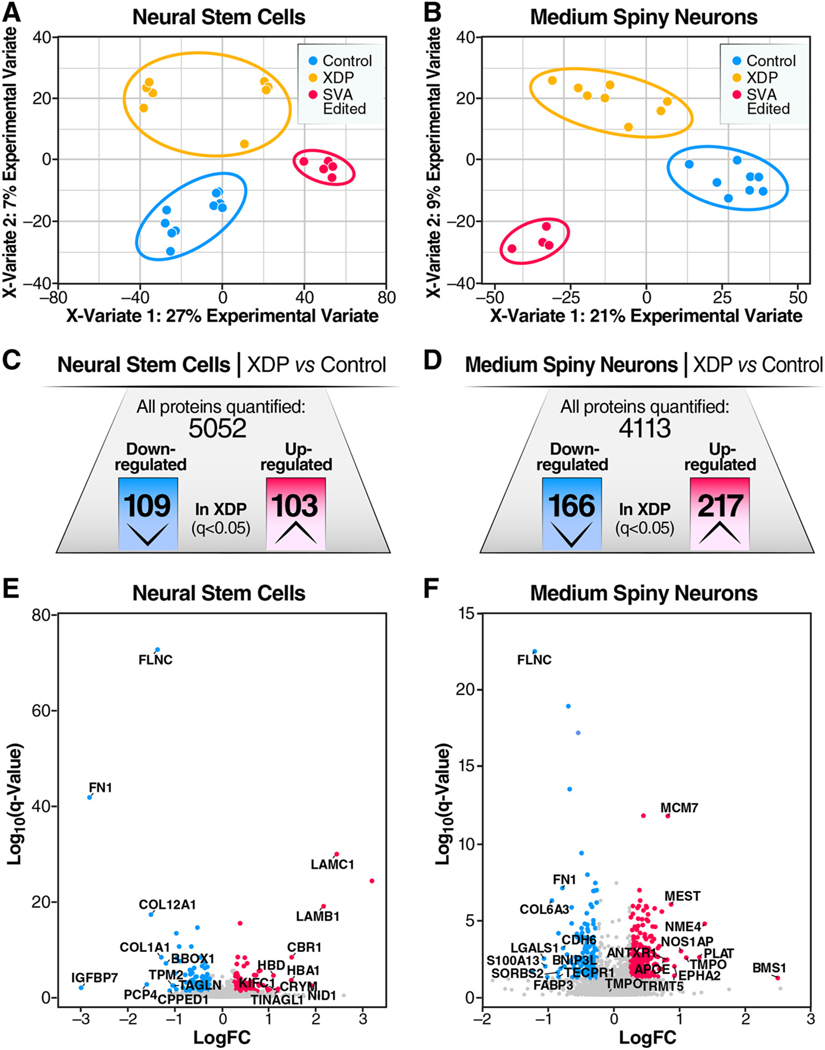
Proteomic analysis of XDP, control and SVA-edited NSCs and MSNs. *A,* NSC and *B,* MSN partial least squares-discrimination analysis of XDP proteomic data set. *C,* NSC and *D,* MSN protein groups were quantified (with ≥2 unique peptides) from DIA performed on the Orbitrap Eclipse MS and with subsequent DIA data analysis using sample-specific hybrid DDA-DIA spectral libraries. Statistically altered proteins were obtained when comparing XDP patients and controls. *E,* NSC and *F,* MSN volcano plots illustrate the proteins differentially expressed when comparing XDP patients and controls, with significant proteins having a q-value set at 5% and absolute log2 (fold-change) ≥ 0.25. For the NSC dataset, one 14i-NSC replicate was excluded from the proteomic analysis making 24 samples. For the MSN dataset, one replicate of each genotype was excluded from the proteomic analysis, thus yielding 20 samples.

Next, we performed a partial least squares-discrimination analysis using the relative protein abundances (Supplementary Table S4, S5) (Rohart et al., 2017). The NSC samples were clustered separately, based on the XDP genotype, controls, and SVA edited (Fig. 3A). Similarly, MSN samples were clustered separately, based on the XDP genotype, controls, and SVA edited (Fig. 3B). The analysis of significantly altered proteins when comparing XDP patients versus controls was depicted in the volcano plots with the q-value set at 5% and absolute log_2_ (fold-change) ≥ 0.25 (Fig. 3E,F). For NSCs, 212 proteins were significantly changed (upregulated, 103; downregulated, 109) (Fig. 3C, Supplementary Table S6). For XDP MSNs, 383 proteins were significantly changed with 217 upregulated and 166 downregulated proteins (Fig. 3D, Supplementary Table S7).

Top upregulated proteins in XDP MSNs included mesoderm-specific transcript homolog protein, minichromosome maintenance-7, nucleotide diphosphate kinase, carboxyl-terminal PDZ ligand of neuronal nitric oxide synthase protein, tissue-type plasminogen activator, lamina-associated polypeptide 2, isoform alpha, ephrin type-A receptor 2, apolipoprotein E, tRNA (guanine(37)-N1)-methyltransferase, and ribosome biogenesis protein BMS1 homolog (Fig. 3F). Moreover, the comparison of XDP patients and controls revealed that several components of the minichromosome maintenance complex, a key DNA helicase for DNA replication, are up-regulated, along with an increase in the number of regulated components in the differentiated neurons, MSNs. Top downregulated proteins include galectin-1, filamin-C, fibronetin, sorbin and SH3 domain-containing protein 2, tectonin beta-propeller repeat-containing protein 1, protein S100-A13, microtubule associated protein, fatty acid binding protein, and BCL2/adenovirus E1B19kDA protein-interacting protein 3-like (Fig. 3F). Notably, dystonia or Parkinson-related proteins are altered in the XDP MSNs proteome, including ATP1A3, COL6A3, GBA, GIGF2, HTRA2, MAP2, SNCA, and TORAIP2. Members of the TFIID complex (TAF1 is a component) are altered in expression, such as BTAF1. BTAF1 is upregulated in XDP vs control MSNs. We did not detect TAF1 in the proteomic analysis. We also evaluated the levels of TAF1 protein and found the levels in XDP MSNs were similar or increased to those in controls with two isoforms detected (Supplementary Fig. 4 A). ICC analysis of TAF1 levels in the XDP and control MSNs were similar (Supplementary Fig. 4B). The XDP CCCTCT repeat is subject to somatic expansions that correlate with the age of onset (Bragg et al., 2017). XDP proteome has changed in the expression of DNA mismatch protein 2, a known modifier of disease onset (Laabs et al., 2021; Trinh et al., 2023; Westenberger et al., 2019). Neurofilament light and medium chain were decreased in the XDP MSNs proteome. Neurofilament light chain levels are significantly elevated in XDP plasma (Al Ali et al., 2021). Numerous RNA binding and processing proteins have altered expression in the XDP proteome (Supplementary Table S7).

**Fig. 4.**
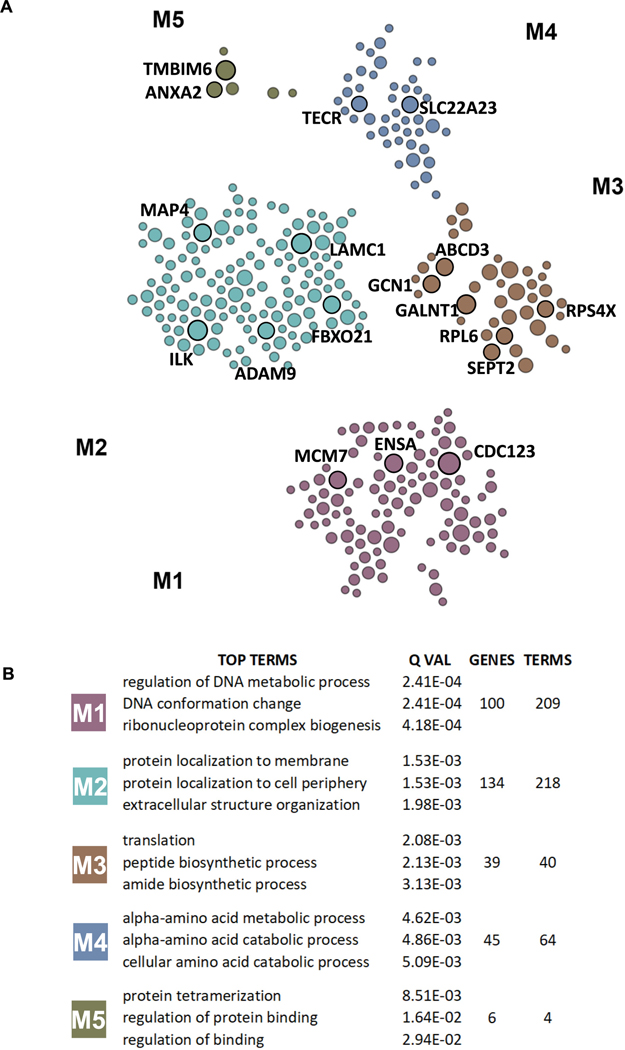
Functional module analysis of XDP MSNs. *A,* and *B,* The biological processes in the list of 383 proteins significantly altered when comparing XDP and control were characterized by using caudate nucleus functional networks at HumanBase (https://hb.flatironinstitute.org/module). These networks represent 343 of 383 proteins and their interactions in biological processes and pathways active in caudate nucleus.

Next, we used the functional model detection method on the proteins altered in the XDP vs control MSNs (HumanBase resource; https://hb.flatironinstitute.org/module). This method clusters genes by their connectivity in a tissue-specific functional network and finds enriched GO terms for each of the gene clusters. We identified five modules in caudate nucleus (Fig. 4A) that include regulation of DNA metabolic process, protein localization to membrane, translation, α-amino acid metabolic process, and protein tetramerization (Fig. 4B). Key proteins within each module are noted (Fig. 4A) and summarized in Supplementary Table S8.

### 2.3. WGCNA analysis of the MSNs proteome identified modules relevant to XDP

**Fig. 5.**
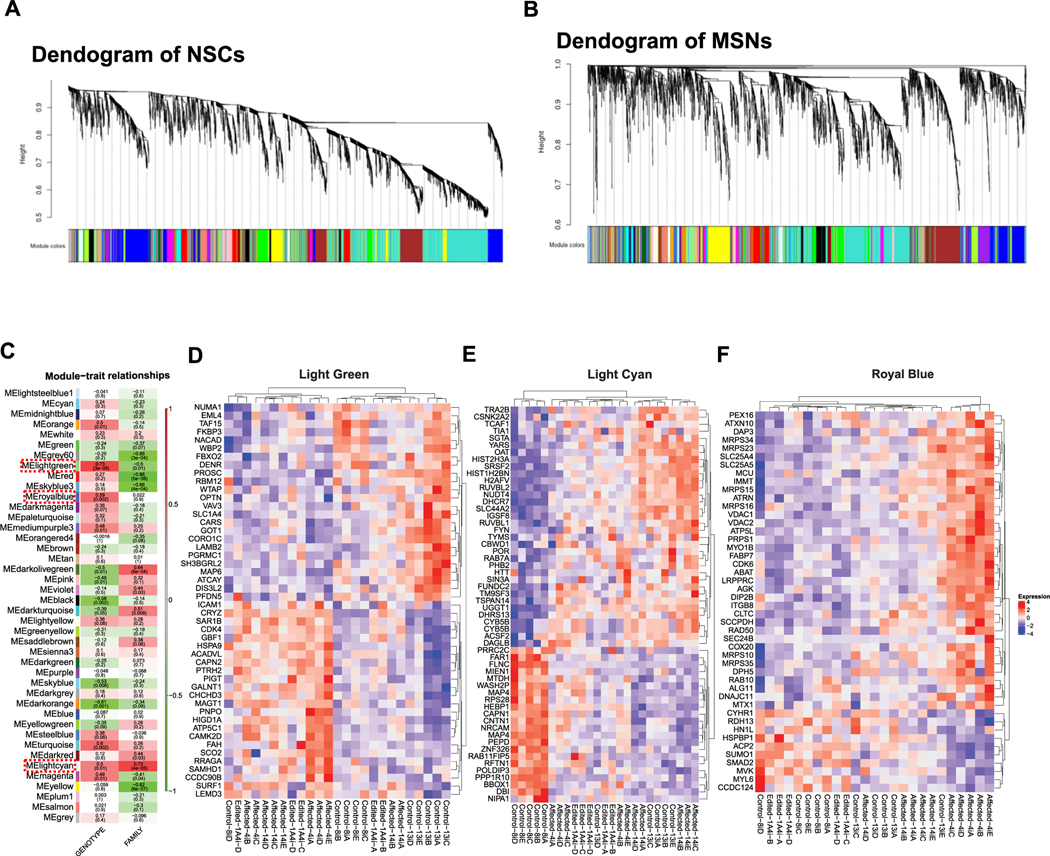
Weighted correlation network analysis of XDP, control and SVA-edited NSC and MSN proteomes. *A,* NSC and *B,* MSN dendrogram of proteins clustered generated using unsupervised hierarchical clustering of all proteins in the entire proteomic data set on the basis of topological overlap, followed by a branch cutting process. Identified modules are defined by colors. *C*, Module-trait relationships for MSN proteomic data set revealed 41 modules. For each module, correlation coefficients are indicated on the top with corresponding *P*-values in the round brackets below. *D,* Light green, *E,* light cyan and *F,* royal blue modules. Heatmap of the proteins present in the MSN modules. (For interpretation of the references to colour in this figure legend, the reader is referred to the web version of this article.)

WGCNA analysis was used to analyze the entire NSC (24 samples) and MSN (20 samples) proteomic data sets to uncover disease-associated network modules, which are clusters of co-regulated proteins that reflect shared functions or cellular components (Langfelder et al., 2016; Langfelder and Horvath, 2008; Yip and Horvath, 2007; Zhang and Horvath, 2005). The construction of a protein co-expression network using normalized protein abundances across all XDP patients, controls, and SVA-edited samples identified 23 and 41 distinct modules for NSCs and MSNs, respectively (Fig. 5A,B, Supplementary Table S9). To determine which modules were relevant to XDP genotype, we computed the biweight mid correlation between each module eigenprotein and the XDP status of the samples. We observed modules derived from the analysis of XDP NSC modules did not reveal genotype-specific alterations (Supplementary Fig. 5), whereas modules produced from the analysis of XDP MSNs showed three modules significantly correlated with XDP: light green, light cyan, and royal blue (Fig. 5C-F).

**Fig. 6.**
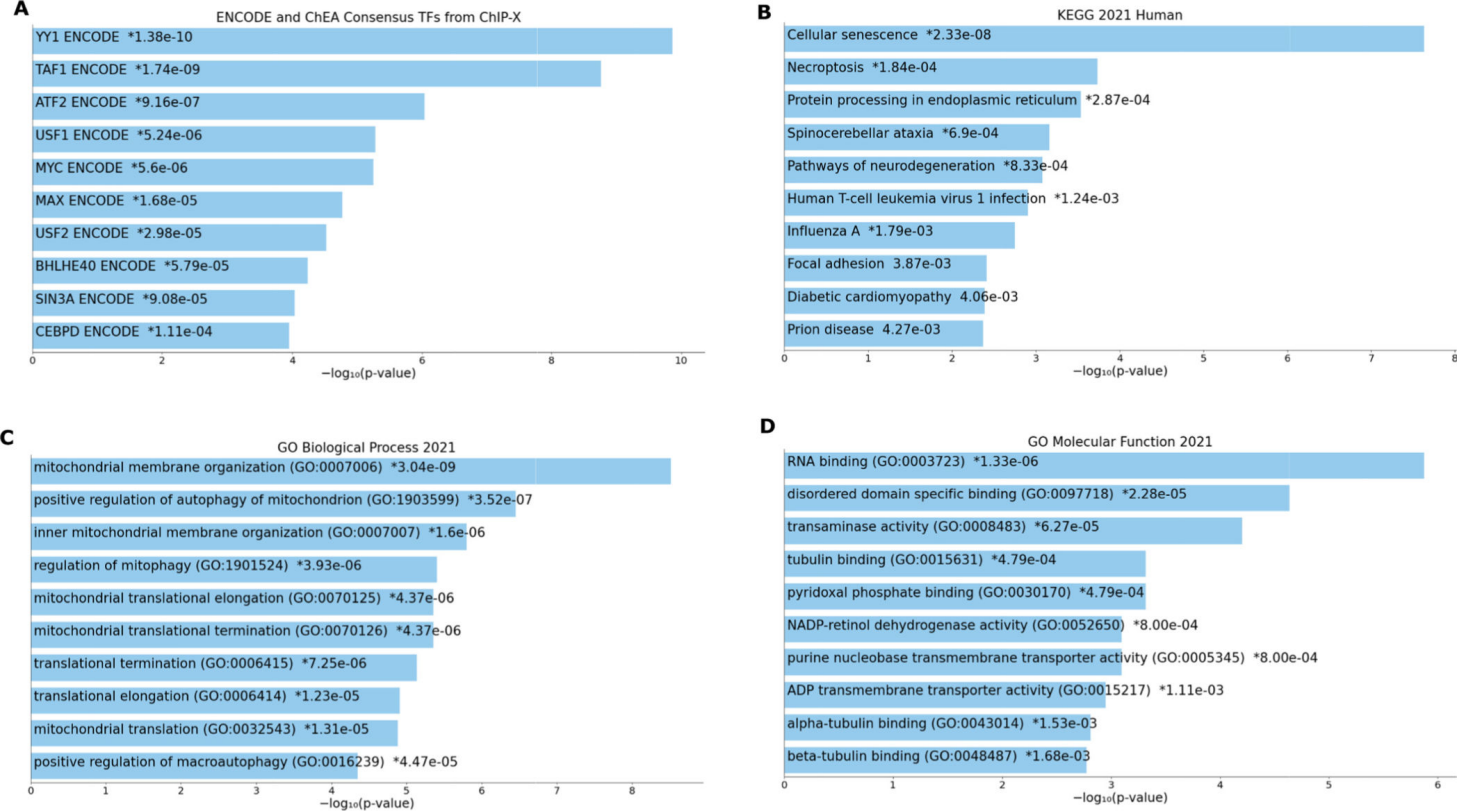
Enrichr analysis of XDP MSN modules identified pathways relevant for XDP neuropathogenesis. Enrichment analysis using *A*, ENCODE and ChEA Consensus TFs from ChiP-X, *B*, KEGG 2021 Human, *C*, GO Biological Process 2021, and *D*, GO Molecular Function 2021 of the MSN modules - light green, light cyan and royal blue combined. (For interpretation of the references to colour in this figure legend, the reader is referred to the web version of this article.)

We combined the three modules and analyzed using Enrichr, a web-based gene list enrichment analysis tool. First, we aimed to characterize the transcription factors (TFs) that are most active using ENCODE and ChEA Consensus TFs (Fig. 6). We found that TAF1 was one of the top enriched TFs, along with YY1, ATF2, USF1 and MYC. Notably, YY1 encodes the protein yin and yang 1, a zinc-finger TF known to be important in central nervous system myelination by interacting with dystonia-associated gene THAP1 (Baumann et al., 2021; Domingo et al., 2021; Yellajoshyula, et al., 2022; Zorzi et al., 2021). Subsequent functional enrichment analysis using database resources, such as BioPlanet 2019, Elsevier Pathway Collection and KEGG, demonstrated that neurodegenerative diseases related pathways, such as HD, spinocerebellar ataxia (SCA), cellular senescence, mitochondrial function, and RNA binding metabolism, are highly enriched in the three MSN WGCNA modules (Fig. 6).

### 2.4. WGCNA light cyan module in XDP

**Fig. 7.**
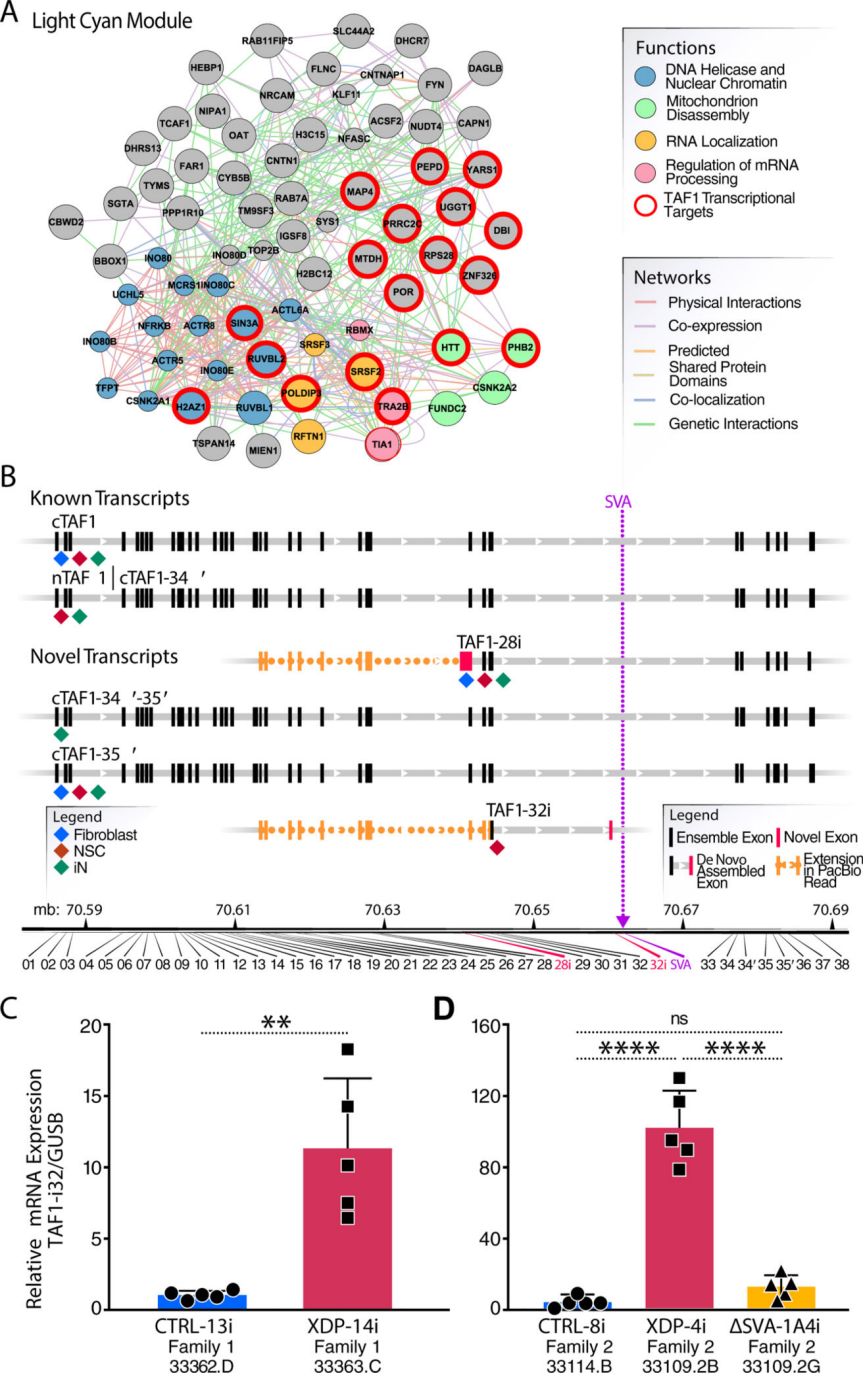
Hub connectivity of light cyan module proteins. *A,* Genemania network analysis of light cyan. All module protein members are in black. Identified functions are defined by colored circles and network connectivity by colored edges. TAF1 transcriptional targets are defined by a red circle. *B,* Genomic segment associated with XDP. XDP is caused by a 13-marker haplotype clustered in noncoding regions within and around TAF1. A ~ 2.6-kb SINE-VNTR-Alu-CCCTCT (SVA)-type retrotransposon in intron 32 of TAF1 has a variable number of hexameric repeats among XDP patients with an increasing repeat number strongly correlated with earlier age at disease onset. Among the known TAF1 isoforms, the canonical transcript (cTAF1) and the neuron-specific isoform of nTAF1, which differs from cTAF1 by incorporation of 6 bp derived from an alternative exon 34. The novel transcripts include one isoform, annotated as “TAF1–32i” that was composed of canonical exon 32 spliced to a cryptic exon in intron 32 that terminated to the SVA. *C,* and *D,* Characterization of TAF1-intron 32 transcript in MSN. qPCR results of MSN derived from XDP patients (*n* = 5), matched controls (n = 5) and SVA edited (n = 5) normalized to GUSB for family 1 (*C*) and family 2 (*D*). Unpaired *t*-test and one-way ANOVA with multiple comparisons were performed for *C* and *D* respectively. **P ≤ 0.01, ****P ≤ 0.0001, ns, not significant. (For interpretation of the references to colour in this figure legend, the reader is referred to the web version of this article.)

Next, we used Genemania to visualize the light cyan module network (Fig. 7A). The proteins clustered into functions that included DNA helicase and nuclear chromatin assembly, mitochondrial disassembly, RNA location, and mRNA processing. Notably, the HTT protein is part of the network. HTT has been linked to TAF1 expression through RNA binding protein SRSF6 (Hernandez et al., 2020). The light cyan module also includes ataxin-10, a protein that causes SCA10 disease when the CAG repeat is expanded in the ataxin-10 gene. Identification of RNA metabolism proteins SRSF2, SRSF3, TRA2B, POLDIP3 and RFTN (Fig. 7A) was consistent with mechanisms relevant to XDP. Interestingly, four of these proteins (i.e., RNPS1, BUD31, SRSF5, TRA2B) are involved in regulation of mRNA splicing. These four proteins interact and form a network. Abnormal RNA processing mechanisms, including splicing and intron retention, are proposed as pathological mechanisms for XDP (Al Ali et al., 2021; Aneichyk et al., 2018; Bragg et al., 2017; Capponi et al., 2020; Herzfeld et al., 2013; Ito et al., 2016; Petrozziello et al., 2020a). Neuronal-specific micro-exon splicing of TAF1 mRNA is directly regulated by SRRM4/nSR100 (Capponi et al., 2020). Since we identified TAF1 as a top enriched TF (Fig. 7), we highlighted with a red circle the genes that are TAF1 transcriptional targets (Fig. 7A). We concede that TAF1 is a subunit of the TFIID complex involved in transcription initiation by RNA polymerase II and has many targets. To date, the differential sensitivities of TAF1 targets in MSNs are unknown.

Aberrant *TAF1* transcription, characterized by alternative splicing and intron retention in the proximity of the SVA insertion, occurs in XDP iPSCs and NSCs (Aneichyk et al., 2018). Previous work on XDP iPSC directly differentiated into cortical neurons using NGN2 induction had undetectable aberrant splicing (Aneichyk et al., 2018). Therefore, we tested if the XDP MSNs model had the TAF1–32i transcript that comprises the canonical exon 32 spliced to a cryptic exon in intron 32 that terminated 715 bp 5′ to the SVA (Fig. 7B). Strikingly, iPSC-derived MSNs from both XDP patient cells had higher levels of TAF1–32i transcript than controls (Fig. 7C, D). As expected, removal of the SVA insertion corrected this aberrant slicing (Fig. 7D). Therefore, we find aberrant expression of TAF1 intron 32 in XDP MSNs.

**Fig. 8.**
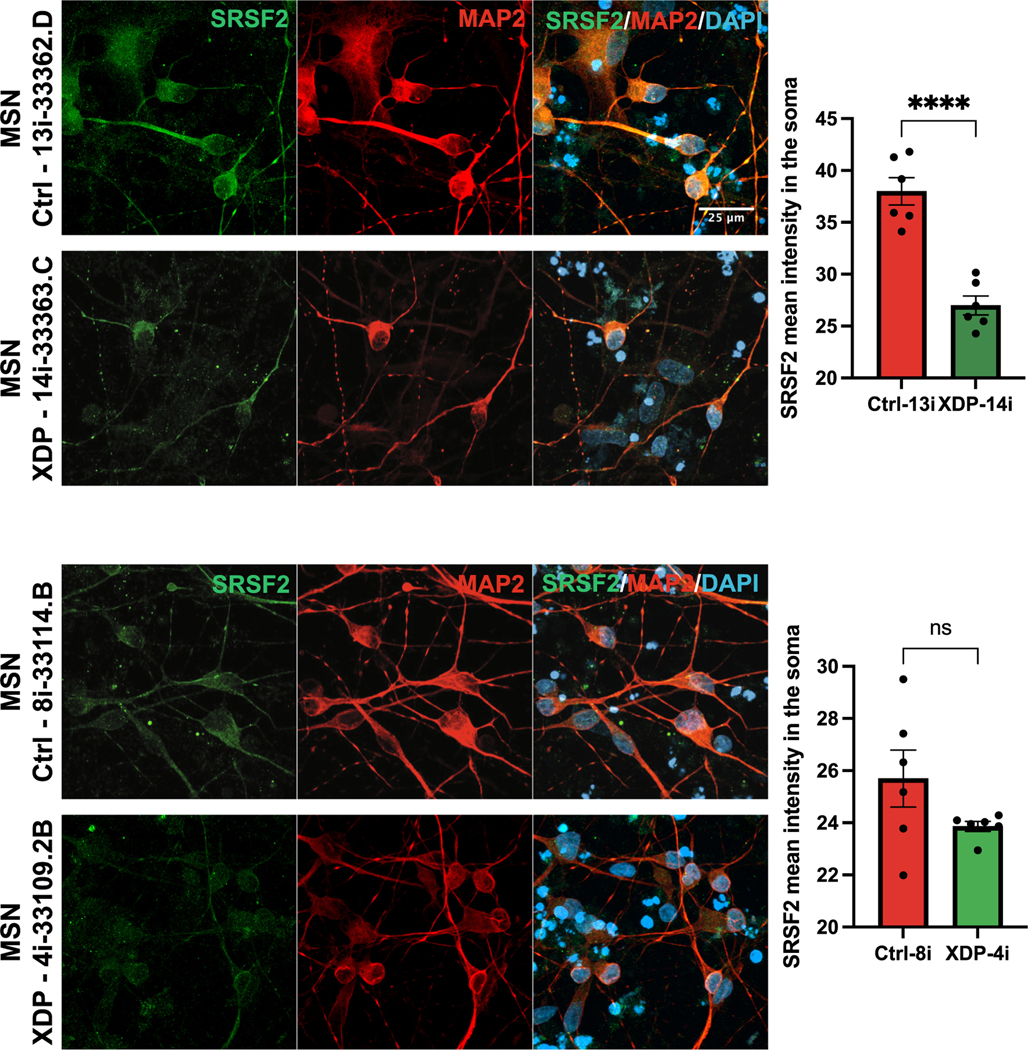
Characterization of SRSF2 expression. MSNs derived from XDP patients and matched controls were immunostained with SRSF2. Scale bars: 25 μm. Quantification of SRSF2 levels in the neuronal soma using MAP2 and DAPI as counterstain. To quantify the levels of SRSF2, *N* = 12 fields were captured in 3 individual wells. Using MAP2 as ROI and substracting the nuclear area, the mean of SRSF2 fluorescence intensity was quantified. *P* values were calculated using t-test ***p ≤ 0.001. Error bars as standard error of mean.

Given the altered splicing in the XDP MSNs, we evaluated the localization of the identified RNA metabolism protein SRSF2. SRSF2 is involved in spinal muscular atrophy and altered splicing of survival motor neuron exon 7, which negatively affects splicing (Wee et al., 2014). SRSF2 localization and levels are altered in Wiskott-Aldrich syndrome, a disease that has widespread altered splicing (Yuan et al., 2022). In control MSNs, we found that SRSF2 was localized to the soma, axons, and dendrites (Fig. 8). In XDP MSNs, the levels of SRSF2 were lower and its localization, when detected, was in the soma (Fig. 8). We used MAP2 to show the health of the neuronal culture morphology, neurite morphology and the dendrites. Loss of MAP2 occurs before cell death (DeGiosio et al., 2022). Our results suggest RNA splicing is altered in XDP and proteins SRSF2, SRSF3, TRA2B, POLDIP3, and RFTN should be further investigated in XDP. Other relevant RNA splicing proteins that were differentially expressed in the XDP proteome include DHX15, DHX9, FUS, GEMIN5, HRNPU, NOVA, PAPOLA, PRPF6 SART3, SRSF5 and RALY, and U2AF1L5 (Supplementary Table S8).

### 2.5. WGCNA royal blue and light green modules in XDP

**Fig. 9.**
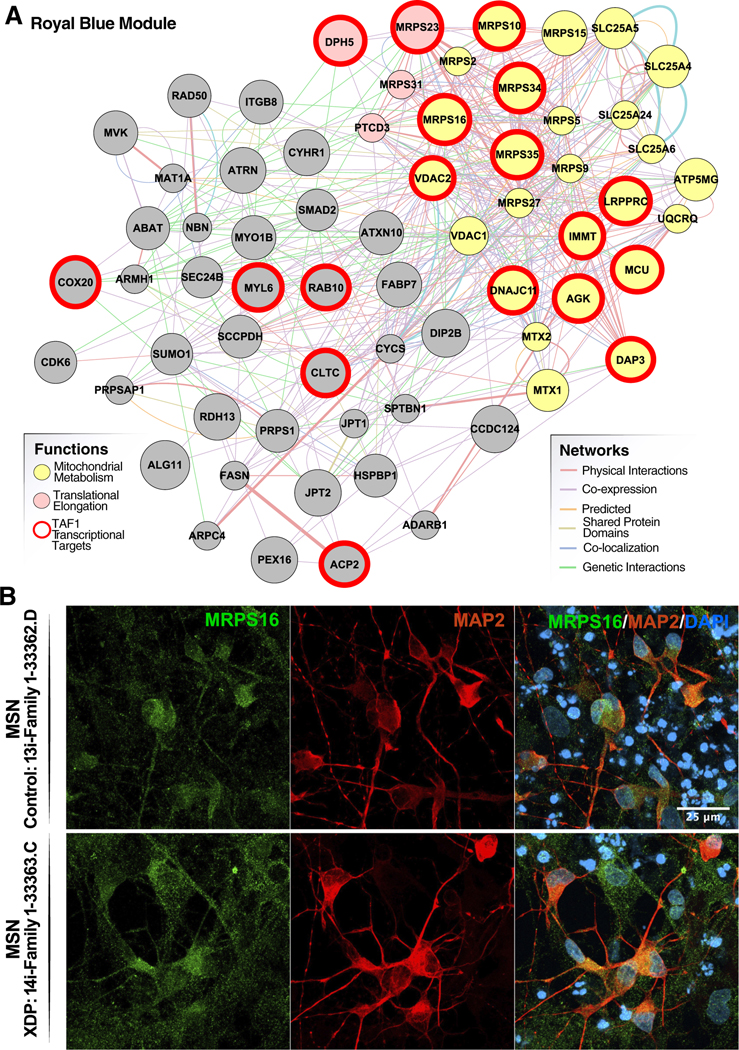
Hub connectivity of royal blue module proteins. *A,* Genemania network analysis of royal blue. All module protein members are in black. Identified functions are defined by colored circles and network connectivity by colored edges. TAF1 transcriptional targets are defined by a red circle. *B,* MSN derived from XDP patients, and matched controls were immunostained with MRPS16. Scale bars: 25 μm. (For interpretation of the references to colour in this figure legend, the reader is referred to the web version of this article.)

Genemania was used to visualize the royal blue module network (Fig. 9A). The proteins clustered into functions that included mitochondrial metabolism and translational elongation. We highlighted with a red circle the genes that are TAF1 transcriptional targets (Fig. 9A). The network contains proteins involved in mitochondrial translation (mitochondrial ribosomal proteins (MRPs) and DAP3), mitochondrial biogenesis (IMMT, DNAJC11, and HSPA9), and mitochondrial calcium ion transport (VDAC2, PHB2, and MCU). Interestingly, we found that Leu-rich PPR motif-containing protein, a factor known to be crucial for mitochondrial mRNA stability (Fig. 9A), is also included. The impairment of the mitochondrial compartment is illustrated by the identification of several MRPs. We found using immunocytochemistry that the levels of MRPS16 are slightly higher in XDP MSNs than controls (Fig. 9B), which may indicate an abnormal synthesis of mitochondrial proteins. Genemania visualization of the light green module highlights the regulation of cell-cycle G1/S phase (cellular senescence), mitochondrial inner membrane, and organelle disassembly (Supplementary Fig. 6).

### 2.6. Drugs predicted to normalize XDP proteomic signature

**Fig. 10.**
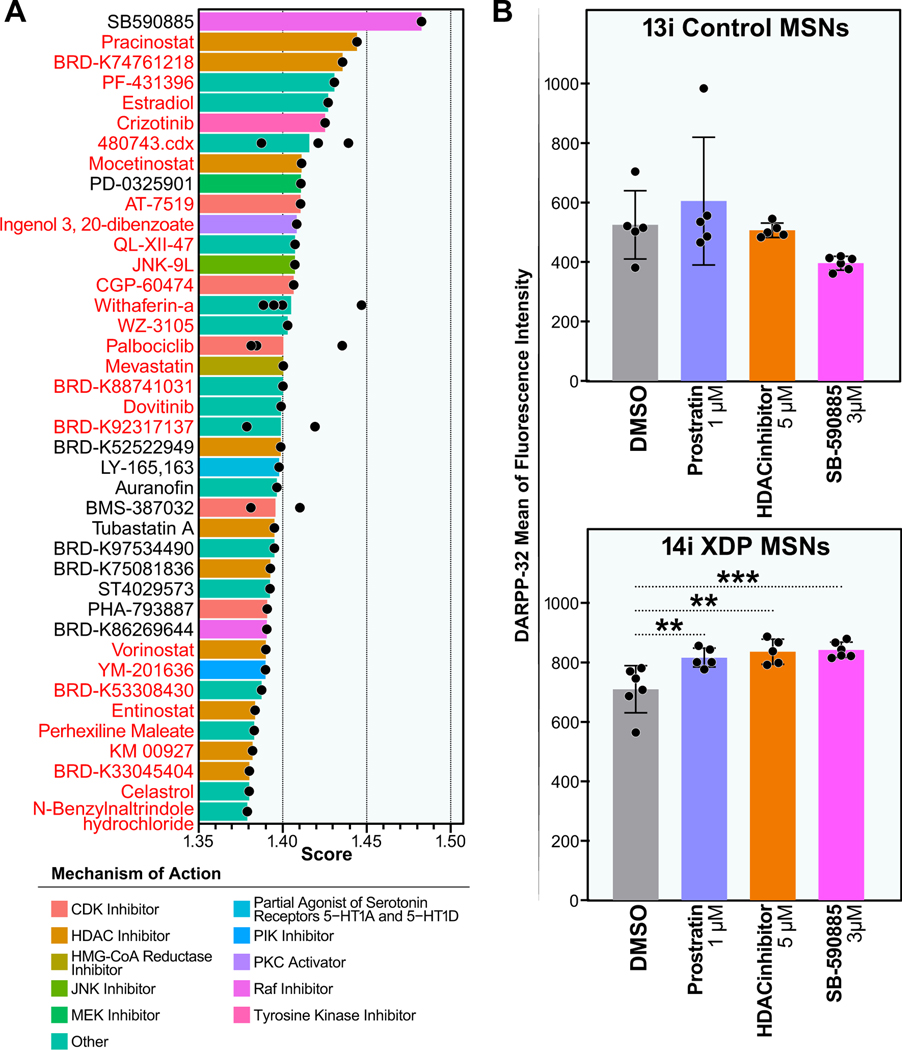
Predicted drugs that normalize XDP proteomic signature. *A*, Top 40 molecules that are predicted to normalize the proteomic XDP signature. The mechanism of action of the molecules are shown below. Highlighted in red show benefit in HD models. *B*, Effect of the drug treatment on XDP MSNs maturation. Evaluation of PKC activator, prostrastin, HDAC inhibitor, and Raf inhibitor, SB-590885 treated with the indicated concentration for 7 days in Synaptajuice B during differentiation of 13i control and 14i XDP MSNs. Quantification of DARPP-32 levels from immunostaining of 13i-Ctrl and 14i-XDP treated MSNs. Cell lines were treated with SB-590885 (*N* = 6 wells, at 3 μM in DMSO), prostratin (N = 6 wells, at 1 μM in DMSO), HDACi inhibitor (N = 6 wells, at 5 μM in DMSO), and DMSO (N = 6 wells) as a control. To quantify the effect of drugs treatment on DARPP-32 level, *N* = 16 fields were captured in individual well. The black circle symbol represents the mean of DARPP-32 fluorescence intensity in individual well. P values were calculated using One-Way Anova Dunnett’s multiple comparisons test, **p ≤ 0.01, and ***p ≤ 0.001. Error bars as standard deviation. (For interpretation of the references to colour in this figure legend, the reader is referred to the web version of this article.)

There is an urgent need to find a treatment for XDP. As shown in Fig. 10A, we used bioinformatics (LINCS) to predict drugs that will reverse the XDP MSN proteomic signature (Supplementary Table S10) (Stathias et al., 2020). These candidates include CDK, HDAC, HMG-CoA reductase, MEK, JNK, Raf and tyrosine kinase inhibitors. Several are effective in HD model systems (in red, Fig. 10). Another interesting drug class predicted includes protein kinase C activators. These have been tested in Phase I and II human trials for Alzheimer’s disease and AIDS (Gutierrez et al., 2016; Jiang et al., 2019; Visser et al., 1988). We evaluated three of the drug classes in the XDP MSNs. Intererestingly, we found PKC activator (prostratin), HDACi inhibitor and Raf inhibitor (SB-590885) increased the levels of DARPP-32 in the XDP MSNs while controls were unchanged (Fig. 10B).

## 3. Discussion

In this study, we characterized the highly specialized and unique proteome of MSNs derived from human patient cells, matched controls, and isogenic SVA-edited cells from two XDP families. To uncover the proteomic changes that occur in progenitor and mature neural cells, we differentiated iPSCs into NSCs and MSNs. The deep proteome coverage for NSCs and MSNs achieved by the DIA-MS workflow allowed identification and quantification of 5052 and 4113 unique protein groups (≥ 2 unique peptides, FDR ≤ 0.01), respectively. Using WGCNA, we observed that NSC samples showed no genotype-specific alterations despite the presence of the XDP haplotype, including the SVA insertion in the TAF1 gene. On the other hand, the analysis of MSN proteome using WGCNA revealed three modules significantly correlated with XDP: light green, light cyan, and royal blue. The analysis of the three modules combined identified TAF1, YY1, ATF2, USF1, and MYC as top enriched TFs based on ENCODE and ChEA Consensus TFs from ChiP-X (Enrichr). The YY1 gene encodes the zinc-finger TF protein yin and yang 1 that interacts with the dystonia-associated gene THAP1 (Baumann et al., 2021; Domingo et al., 2021; Yellajoshyula, 2022; Zorzi et al., 2021) and activates the myelination gene expression program centered on the TF EGR2 (He et al., 2010), thus highlighting the crucial role of YY1 in central nervous system myelination. Interestingly, YY1 de novo mutations cause Gabriele-de Vries syndrome, a form of syndromic intellectual disability characterized by a complex movement disorder, including ataxia and progressive dystonia along with prominent laryngeal involvement. The latter two symptoms are similar to those found in XDP.

The location of the XDP haplotype in and around *TAF1* suggested that the regulation of TAF1 expression may be critical for XDP pathology. Expression of the XDP-associated aberrant transcript TAF-*32i* is greater in XDP patients than controls (Al Ali et al., 2021; Aneichyk et al., 2018; Pozojevic et al., 2022). Our analyses demonstrated that the MSNs derived from XDP patients retain the molecular signature of TAF-*32i* expression. Interestingly, we showed that deletion of the SVA insertion led to a significant decrease of TAF-*32i* expression in MSNs, similar to the control level. However, the exact molecular mechanisms of the abnormal level of TAF-*32i* in XDP diseases remain unclear. The SVA insertion in an intron of the *TAF1* gene contains a polymorphic hexanucleotide repeat (CCCTCT)n, causing the formation of secondary RNA structures known as G-quadruplexes (Bragg et al., 2017). Those structures can lead to aberrant DNA transcription, repeat-associated non-ATG (RAN) translation, mRNA processing, transport, and translation (Cammas and Millevoi, 2017). Of note, the aforementioned abnormal activities strongly overlap with the functions of the protein targets observed in the light cyan and royal blue modules. We hypothesize that the SVA insertion leads to abnormal expression of TAF-*32i* in XDP MSNs and thus exacerbates the aberrant activities of G-quadruplexes by targeting proteins involved in RNA metabolism, including SRSF2, POLDIP3, TRA2B, and TIA1. We showed that SRSF2, an RNA-binding protein involved in splicing of mRNA precursors, was significantly downregulated in XDP MSNs. This suggests that TAF1 dysregulation impairs the recruitment of the core spliceosome. Future studies need to establish the molecular mechanisms by which TAF1 defects alter the SRSF2 RNA-binding activities and which genes are predominantly affected.

We discovered several important mitochondrial pathways, such as mitochondrial translation (mitochondrial ribosomal proteins (MRPs) and DAP3), mitochondrial biogenesis (IMMT, DNAJC11, and HSPA9), and mitochondrial calcium ion transport (VDAC2, PHB2, and MCU), were altered in XDP MSNs. These results strongly suggest that mitochondrial dysfunction is one of the key drivers for XDP neuropathology. The impairment of the mitochondrial compartment is illustrated by the identification of several MRPs. Interestingly, the Leu-rich PPR motif-containing protein was also a TAF1 transcriptional target. Our results suggest further analysis of the interplay between the aberrant activities of TAF-*32i* expression and mitochondrial function should be carried out.

In addition, we demonstrated that cellular mechanisms related to neurodegenerative diseases, including HD and SCA, are enriched in the XDP MSNs proteome. The identification of cellular senescence features in the XDP MSNs proteome is consistent with numerous studies reporting cellular senescence as one of the drivers for neurodegenerative diseases (Bussian et al., 2018; Chinta et al., 2018; Mendelsohn and Larrick, 2018; Zhang et al., 2019). Cellular senescence is a prominent feature of HD MSNs derived from patient iPSCs (Tshilenge et al., 2023; Voisin et al., 2020).

The prediction of drugs that reverse the XDP MSNs proteome toward control is significant and the streamlining of therapeutic candidates for XDP patients is urgently needed. One class of drugs predicted are HDAC inhibitors. Notably, the HDAC inhibitor, sodium phenylbutyrate-taurursodiol, was evaluated in Phase II clinical trials for amyotrophic lateral sclerosis and provided 6.5 months more survival than a placebo (Paganoni et al., 2021). Protein kinase C activators were predicted to reverse the XDP proteome signature, and this class of drugs has been tested in Phase I and II human trials for AD and AIDS (Gutierrez et al., 2016; Jiang et al., 2019; Visser et al., 1988).

There are several limitations of our studies. The MSN model we used is developmental and does not represent human adult MSNs. The MRPs are a large family of proteins involved in mitochondrial translation and further studies are needed to understand their role in XDP. Finally, the pathogenesis of XDP involves multiple cells types in the brain and we limited our studies to MSNs.

The selective loss of MSNs within the striatum in XDP is similar to the neurodegeneration observed in HD, suggesting common molecular mechanisms (Tshilenge et al., 2023). Strikingly, we found that proteins associated with the HD signature are enriched in the XDP MSNs WGCNA data set (light cyan, royal blue and light green modules), including HTT, SIN3A, CAPN2, CAPN1, VDAC1, and VDAC2. These observations suggest that the aberrant level of the alternative splicing isoform TAF1-*32i* triggers a transcriptional dysregulation of genes that overlap with the HD proteome. Our quantitative unbiased proteomics analysis using human-derived neural cells from XDP patient iPSCs represents a useful resource for the XDP communities for further understanding the neuropathogenesis of XDP. These findings may contribute to the identification of new molecular markers and pathophysiologic drivers for XDP and thus promote the development of innovative therapeutics for this disease.

## 4. Methods

### 4.1. Human iPSC-derived NSC cultures

XDP patients, matched controls, and SVA-edited iPSCs were maintained in mTeSR^™^1 (STEMCELL Technology, 85850) medium at 37 °C and with 5% O_2_ before differentiation. The reprogramming of XDP cells and controls has been previously described (Ito et al., 2016) as well as CRISPR/Cas9-based generation of the edited clones (Aneichyk et al., 2018). iPSCs were grown on Matrigel (Corning, 354230) and passaged when they reached 70–80%. The genotype of each line was confirmed by PCR. To induce iPSC toward a neuroepithelial fate, we used a neural rosette differentiation approach as described (Tshilenge et al., 2023). NSCs were passaged when the cell cultures became confluent. We cultured the cells in 6-cm plates at high density. SOX2, PAX6, and NESTIN staining of NSCs validated the cell type. Details of the immunocytochemistry are provided below.

### 4.2. MSN differentiation

Activin A (25 ng/mL, PeproTech, AF-120–14E)-generated XDP patients, matched controls and SVA-edited NSCs were used to prepare MSNs. Nunc six-well plates were treated with poly-D-lysine hydrobromide (1 mL, 100 μg/mL by Sigma Aldrich, P6407) and incubated (37 °C and 5% CO_2_) overnight (ON). Detailed methods are as described (Tshilenge et al., 2023). MSNs were prepared according to Kemp et al. (Kemp et al., 2016). For the first 7 days, cells were treated with Synaptojuice A, and on day 8, they were treated with Synaptojuice B for 14 days. Half-medium changes were performed until day 21. Rabbit anti-MAP2 (Millipore, AB5622, 1:100), rabbit anti-GABA (Sigma, A2052, 1:100), and rabbit anti-TUBB3 (Cell Signaling, 5568, 1:100) staining of MSNs validated the cell type.

### 4.3. Genotyping of the XDP patient lines

To identify SVA retrotransposon, long-range PCR was adapted from (Aneichyk et al., 2018). Briefly, primers were designed in the flanking region of the SVA retrotransposon, based on the genomic sequences obtained from XDP patients (NCBI Accession Number AB191243). Sequences of the primer set flanking SVA retrotransposon are XDP-16153 F: 5′-GTTCCATTGTGTGGTTGT-ACCAGCGTTTGTTC-3′, XDP-19345R: 5′-CACATGAAAAGATGCCCAACATCATTAGC-CATTAG-3′ (Integrated DNA Technologies, Coralville, IA). The long-range PCR amplicons were electrophoresed on 1% agarose gel to detect a 3229-bp (corresponding to the ~2.6-kb SVA insertion) or 599-bp product (without a SVA insertion).

### 4.4. Quantitative real-time PCR of human XDP MSNs

TAF1-intron 32 expression was evaluated by RT-qPCR. Briefly, RNA was isolated from MSNs using ISOLATE II RNA Mini Kit (Bioline, BIO-52072). cDNA was prepared from 300 ηg of RNA in a total reaction volume of 20 μL using the SuperScript IV VILO Master Mix cDNA kit (Thermo Fisher Scientific, 11756050). Subsequently, a nested PCR for TAF1-intron 32 was performed using cDNA as a template to run 15 cycles of PCR with 0.5 μM of TAF1-intron 32 primers (Integrated DNA Technologies) and Phusion Hot Start Flex 2× Master Mix (New England Biolabs, M0536L) in a 50-μL total reaction. TAF1-intron 32 primers target exon 32 (Forward: 5′-ACATCTCCAAGCACAAGTATCA-3′) and intron 32 (Reverse: 5′-GTAATGTACCAATATAAATTT-CCTGGTTT-3′). Cycling conditions for the three-step amplification are as follows: 98 °C for 30 s; 15 cycles of 98 °C for 10 s, 61 °C for 30 s, 72 °C for 30 s, and 72 °C for 5 min. Then, the nested PCR products were cleaned up using DNA Clean and Concentration (Zymo Research, D4004) and eluted in 20 μL. For quantitative PCR, TaqMan Fast Advanced Master Mix (Thermo Fisher Scientific, 4444557) was used in a 10-μL reaction volume, on the QuantStudio™ 6 Pro Real Time PCR System (Thermo Fisher Scientific). The cycling conditions consisted of 40 cycles of 1 s at 95 °C and 20 s at 60 °C each, followed by dissociation curve analysis, using custom primers i32-FAM (Thermo Fisher Scientific, Custom ID #AJWR28J, 4,441,114) and GUSB-VIC-PL (Thermo Fisher Scientific #Hs00939627_m1, 4,331,182). The ΔCt value was calculated by subtracting the Ct for the endogenous control gene GUSB from the Ct value of the gene of interest. Relative quantification was performed using the ΔCt method and expressed as a fold-change relative to the control by calculating 2^-ΔCt^.

### 4.5. Cell immunofluorescence of human NSCs and MSNs

Cells were fixed using 4% paraformaldehyde (Sigma, 158,127) in 0.1 M phosphate-buffer saline (PBS), pH 7.4 (Corning, 21–040-CV), for 30 min. After three washes in cold PBS, cells were permeabilized and blocked for 1 h at RT using 0.1% Triton X-100 (Thermo Fisher Scientific, 28313) and 4% normal donkey serum (Jackson Immuno Research, 017–000–121) in PBS. Primary antibodies were added in the presence of a blocking buffer overnight at 4 °C. Secondary antibodies (1:500) were added after three PBS washes in the blocking buffer at RT for 1 h. The following primary antibodies were used for the immunofluorescence studies: rabbit anti-SOX2 (Cell Signaling, 3579S, 1:200), rabbit anti-PAX6 (Biolegend, 19013, 1:100), rabbit anti-NESTIN (Abcam, ab92391, 1:100), rabbit anti-MAP2 (Millipore, AB5622, 1:100), rabbit anti-GABA (Sigma, A2052, 1:100), rabbit anti-DARPP-32 (Cell Signaling, 2306S, 1:200), rabbit anti-TAF1 (Sigma, HPA001075) and rabbit anti-TUBB3 (Cell Signaling, 5568, 1:100). Secondary antibodies were donkey anti-rabbit and anti-mouse IgG, conjugated with Alexa-546 (Invitrogen, A10040 and A10036) or Alexa-647 (Invitrogen, A-31573 and A-31571). Images were acquired using a Biotek Cytation 5 and Zeiss LSM 980 laser scanning confocal microscope and were prepared using Fiji software (ImageJ). Quantification of SRSF2 levels in the neuronal soma using MAP2 and DAPI as counterstain. To quantify the levels of SRSF2, *N* = 12 fields were captured in 3 individual wells. Using MAP2 as ROI and substracting the nuclear area, the mean of SRSF2 fluorescence intensity was quantified.

### 4.6. Protein extraction for proteomic analysis

The XDP patients, matched controls, and SVA-edited cultures of NSC and MSN were washed three times with cold PBS 1×, pH 7.4 (Corning, 21–040-CV), and total protein lysates were isolated using 300 μL of lysis buffer containing 0.5% sodium dodecyl sulfate in 50 mM of triethylammonium bicarbonate with protease/phosphatase inhibitors (Thermo Fisher Scientific, 78442). The cell lysate was harvested by scraping and transferred directly into a cold 1.5-mL tube and stored at −80 °C.

### 4.7. Western blot analysis

Lysates (10 mg) were prepared in 4× sample buffer (Invitrogen, NP0007) with 0.05 M DTT and run on a 3–8% Tris-acetate gel (Invitrogen, EA0375BOX) at 200 V for 35 min in Tris-acetate running buffer (Invitrogen, LA004) with antioxidant. Transfer was performed overnight, 20 V for 840 min at 4 °C onto a 0.45-μm nitrocellulose membrane (Thermo Scientific, 88018). Membranes were probed with primary antibody to TAF1 (1:200, CCXDP, TAF-1 AK). Membranes were incubated with secondary anti-rabbit HRP (1:3000, Invitrogen, NA934V). Loading control β-actin antibody was used to probe the blot (1:5000, Santa Cruz, sc-47778). Proteins bands were detected by chemiluminescence (Pierce ECL; Thermo Fisher Scientific, 32,106). Image ab (Bio-Rad) was used for densitometry analysis.

### 4.8. Proteomic sample preparation

#### 4.8.1. Chemicals

Acetonitrile (AH015) and water (AH365) were from Burdick & Jackson (Muskegon, MI). Iodoacetamide (I1149), dithiothreitol (DTT, D9779), formic acid (94318–50ML-F), and triethylammonium bicarbonate buffer 1.0 M, pH 8.5 (T7408), were from Sigma Aldrich (St. Louis, MO). Urea (29700, Thermo Fisher Scientific, Waltham, MA), sequencing grade trypsin (V5113, Promega, San Luis Obispo, CA), and HLB Oasis SPE cartridges (186003908, Waters, Milford, MA) were used for the mass spectrometry experiments.

#### 4.8.2. Protein precipitation, digestion and desalting

Protein samples were precipitated with a ProteoExtract Protein Precipitation Kit (539180) from Millipore Sigma (Burlington, MA) as per the manufacturer’s protocol. Samples were resuspended in 50 mM triethylammonium bicarbonate (TEAB). Total protein concentrations were determined with a BCA kit (23227) from Thermo Fisher (Waltham, MA). Aliquots of each sample containing ~100 μg of protein were brought to equal volumes with water. Samples were solubilized with 4% SDS and 50 mM TEAB at pH 8. Proteins were reduced with 20 mM DTT (10 min at 50 °C followed by 10 min at RT) and then alkylated with 40 mM iodoacetamide (30 min at RT in the dark). Samples were acidified to a final concentration of 1.2% phosphoric acid and diluted with seven volumes of S-trap buffer (90% methanol in 100 mM TEAB, pH 8). Samples were then loaded onto the S-trap micro-spin columns (Protifi, Farmingdale, NY) and washed twice with S-trap buffer before adding a solution of sequencing grade trypsin (Promega, San Luis Obispo, CA) in 50 mM TEAB at a 1:25 (w:w) enzyme:protein ratio. After a 1-h incubation at 47 °C, trypsin solution was added again at the same ratio, and proteins were digested overnight at 37 °C. Peptides were sequentially eluted with 50 mM TEAB, 0.5% formic acid (FA) in water, and 50% acetonitrile (ACN) in 0.5% FA. After vacuum drying, samples were resuspended in 0.2% FA in water, desalted with Oasis 10-mg Sorbent Cartridges (Waters, Milford, MA). Samples were vacuum dried again and resuspended in 0.2% FA in water at a final concentration of 1 μg/μL. Finally, indexed retention time standard peptides (iRT; Biognosys, Schlieren, Switzerland) were spiked into the samples according to manufacturer’s instructions.

### 4.9. Mass spectrometric analysis

LC-MS/MS analyses were performed on a Dionex UltiMate 3000 system coupled to an Orbitrap Eclipse Tribrid mass spectrometer (both from Thermo Fisher Scientific, San Jose, CA). The solvent system consisted of 2% ACN, 0.1% FA in water (solvent A) and 98% ACN, 0.1% FA in water (solvent B). Peptides (200 ng) were loaded onto an Acclaim PepMap 100 C18 analytical column (0.1 × 20 mm, 5-μm particle size; Thermo Fisher Scientific) over 5 min at 5 μL/min with 100% solvent A. Peptides were eluted on an Acclaim PepMap 100 C18 analytical column (75 μm × 50 cm, 3 μm particle size; Thermo Fisher Scientific) at 0.3 μL/min using the following gradient of solvent B: 2% for 5 min, linear from 2% to 20% in 125 min, linear from 20% to 32% in 40 min, up to 80% in 1 min, 80% for 9 min, and down to 2% in 1 min. The column was equilibrated at 2% for 29 min (total gradient length = 210 min). Every sample was acquired in DIA mode for the quantitative analysis using the following settings: full MS spectra were collected at 120,000 resolution (AGC target: 3e6 ions, maximum injection time: 60 ms, 350–1650 *m*/*z*), and MS2 spectra at 30,000 resolution (AGC target: 3e6 ions, maximum injection time: Auto, NCE: 27, fixed first mass 200 m/z). The isolation scheme consisted in 26 variable windows covering the 350–1650 m/z range with an overlap of 1 m/z (Supplementary Table S1) (Bruderer et al., 2017).

In addition, one replicate from each XDP patient, matched control, and SVA-edited cultures of NSC and MSN (total = 12 samples) was analyzed in DDA for generating the spectral libraries. Full MS spectra were collected at 240,000 resolution (AGC target: 1.2e6 ions, maximum injection time: Auto, 350–1500 m/z). Precursor ions with a charge state 2–5+ were automatically selected for HCD fragmentation at NCE 28 in the ion trap. MS2 spectra were collected with scan rate set to Turbo (AGC target: 3e4 ions, maximum injection time: 35 ms, scan range: Auto).

### 4.10. Proteomic data analysis

#### 4.10.1. Spectral library generation

DDA-DIA hybrid spectral libraries were generated in Spectronaut (version 14.10.201222.47784; Biognosys, Schlieren, Switzerland) using BGS settings and a human UniProtKB-TrEMBL database (92,931 entries, release 01/2018). Briefly, for the Pulsar search, trypsin/P was set as the digestion enzyme, and two missed cleavages were allowed. Cysteine carbamidomethylation was set as fixed modification, and methionine oxidation and protein N-terminus acetylation were set as variable modifications. Identifications were validation using 1% false discovery rate (FDR) at the peptide spectrum match (PSM), peptide and protein levels, and finally the best 3–6 fragments per peptide were kept. The XDP-NSC library contains 58,855 peptides and 6089 protein groups, whereas the XDP-MSN library contains 43,335 modified peptides and 5092 protein groups. The spectral libraries generated can be found in Supplementary Tables S2 and S3.

#### 4.10.2. DIA data processing and statistical analysis

DIA data was processed in Spectronaut (version 15.1.210713.50606) using the above hybrid libraries. Data extraction parameters were set as dynamic and non-linear iRT calibration with precision iRT was selected. Identification was performed using 1% precursor and protein q-value. Quantification was based on MS2 extracted ion chromatogram (XIC) area, local normalization was applied, and indexed retention time (iRT) profiling was selected. Differential protein expression analysis was performed using paired *t*-test, and *p*-values were corrected for multiple testing using the Storey method (Burger, 2018; Storey, 2002). Protein groups were required to have at least two unique peptides. Finally, protein groups with q-value ≤0.05 and absolute Log2(fold-change) > 0.25 were considered to be significantly altered for the ‘XDP vs control’ comparisons (Supplementary Tables S6, S7).

### 4.11. Data accession

Raw data, complete MS data sets, and spectral libraries have been uploaded to the Center for Computational Mass Spectrometry and to the MassIVE repository at UCSD and can be downloaded using the following link: https://MSV000092344@massive.ucsd.edu (MassIVE ID number: MSV000092344; ProteomeXchange ID:PXD043562).https://massive.ucsd.edu/ProteoSAFe/dataset.jsp?task=f168b6b041ae47a5bbe6ffb63c100b20PXD043562

### 4.12. Clustering analysis

Partial least squares-discriminant analysis of the proteomics data was performed using the package mixOmics in R (version 4.0.2; RStudio, version 1.3.1093) (Rohart et al., 2017).

### 4.13. Functional module analysis of significantly altered proteins in XDP MSNs

To identify the functions enriched in the lists of significantly altered proteins in XDP MSNs, we used the functional module detection method from the HumanBase resource (https://hb.flatironinstitute.org/module). The method clusters genes by their connectivities in a tissue-specific functional network and finds enriched GO terms for each of the gene clusters.

### 4.14. Weighted coexpression network analysis

Protein co-expression network analysis was done utilizing log normalized protein abundance with the WGCNA R package (Langfelder and Horvath, 2008) as described (Gu et al., 2015).

### 4.15. Enrichment and networking analysis

Selected module members were inputted into Enrichr, a web-based gene list enrichment analysis tool (Chen et al., 2013; Kuleshov et al., 2016; Xie et al., 2021). Enrichment terms are scored by p-value, z-score, and combined score, and shown in figures sorted by p-value. Relevant modules were further investigated using Genemania (Warde-Farley et al., 2010).

### 4.16. Comparison with multiple datasets and drug prediction

Enrichment analysis for GO biological processes with differentially expressed proteins (FDR *<* 0.05, logFC *>*0.58) was done utilizing the R package clusterProfiler. Drug prediction was done utilizing the LINCS L1000 characteristic direction signatures search engine (https://maayanlab.cloud/L1000CDS2/#/index) with upregulated and downregulated proteins as input (Duan et al., 2016).

### 4.17. Drug treatment

The drug treatment was performed during the differentiation process of MSNs using 13i-Ctrl and 14i-XDP lines. To produce MSNs, NSCs were plated at 100,000/well in a 96-well plate (MilliporeSigma, Corning, CLS3340). For the first 7 days, cells were treated with Synaptojuice A, and on day 8, they were treated with Synaptojuice B for 7 days. Half-medium changes were performed until day 14. The drugs were diluted in Synaptojuice B to the desired concentration and added to the cells starting on day 8 of differentiation and every time the medium was changed until the cells were harvested.

The following drugs were used: SB-590885 (Tocris, 2650, at 3 μM in DMSO), prostratin (MilliporeSigma, 60857–08–1) at 1 μM in DMSO, and HDACi inhibitor (5 μM in DMSO) (Lai et al., 2017). To evaluate the effect of drugs treatment on MSNs generation, the cells were fixed using 4% paraformaldehyde (Sigma, 158127) for 30 min and then stained using rabbit anti-DARPP-32 (Cell Signaling, 2306S, 1:200). The secondary antibody was donkey anti-rabbit IgG conjugated with Alexa-647 (Invitrogen, A-31573). Images were acquired and quantified using a Biotek Cytation 5.

### 4.18. Experimental design and statistical rationale

XDP iPSCs were derived from two families, with each containing the carriers (XDP-14i-Family 1 and XDP-4i-Family 2) and the respective controls (Ctrl-13i-Family 1 and Ctrl-8i-Family 2). In addition, we included an XDP isogenic line in which the deletion of the SVA was performed (SVA-edited-1A4i-Family 2). XDP iPSCs were differentiated into neural cells (NSCs and MSNs) and grown in parallel with five replicates for each genotype. Hence, *N* = 25 for NSCs and N = 25 for MSNs. Proteomic experiments were conducted on these total 50 samples. Indexed retention time standard peptides (iRT; Biognosys, Schlieren, Switzerland) were spiked into the samples according to manufacturer’s instructions before LC-MS/MS analysis in DDA and DIA modes on the Orbitrap Eclipse Tribrid mass spectrometer. More precisely, one DIA cycle was composed of the acquisition of one MS1 scan, followed by the acquisition of 26 variable windows (18–574 *m*/*z*) covering the full mass range (m/z 350–1650) with an overlap of 1 m/z. DDA and DIA data were used to build sample-specific hybrid spectral libraries, and DIA data for MS2 XIC-based label-free quantification using the generated spectral libraries. To determine significantly altered protein groups, pairwise comparisons were performed using a paired *t*-test for DIA-based quantification and obtained p-values were corrected for multiple testing using the Storey method(Burger, 2018), as described above.

## Supplementary Material

suppl Table 1

suppl Table 2

suppl Table 4

suppl Table 3

suppl Table 5

suppl Table 6

suppl Table 8

suppl Table 9

suppl Table 10

suppl Table 7

suppl material western blots

Suppl Figures 1-6

## Figures and Tables

**Table 1:** XDP patient lines.

Samples for proteomic analysis								

Name	Standard name	Age at collection	Status	Sex	Relationships	Hexameric repeat size	NSCs	MSNs
Ctrl-13i Family 1	MIN13i-33362.D	18	Control	M	Son of proband 33363	Not available	N = 5 at P6	*N* = 5 at P6
XDP-14i Family 1	MIN14i-33363.C	44	Affected	M	Proband 33363	40	N = 5 at P5	N = 5 at P5
Ctrl-8i Family 2	MIN08i-33114.B	34	Control	M	Son of proband 33109	Not available	N = 5 at P7	N = 5 at P7
XDP-4i Family 2	MIN04i-33109.2B	72	Affected	M	Proband 33109	36	N = 5 at P5	N = 5 at P5
SVA-edited-1A4i Family 2	33109–2G-dSVA-1A4i	72	Edited	M	Proband 33109	Not available	N = 5 at P5	N = 5 at P5

Ctrl, Control; M, Male; P, passage; SVA, SINE-VNTR-Alu, XDP, X-linked dystonia-parkinsonism.

## Data Availability

Raw data, complete MS data sets, and spectral libraries have been uploaded to the Center for Computational Mass Spectrometry and to the MassIVE repository at UCSD and can be downloaded using the following link: https://massive.ucsd.edu/ProteoSAFe/dataset.jsp?task=f168b6b041ae47a5bbe6ffb63c100b20 (MassIVE ID number: MSV000092344; ProteomeXchange ID: PXD043562).

## Abbreviations:

ATF2: activating transcription factor 2
CNS: central nervous system
Ctrl: control
DDA: data-dependent acquisition
DIA: data-independent acquisition
EGR2: early growth response 2
FDR: false-discovery rate
GABA: gamma-aminobutyric acid
HD: Huntington’s disease
HTT: Huntingtin
iPSC: induced pluripotent stem cells
Kb: kilobase
KEGG: Kyoto Encyclopedia of Genes and Genomes
LC-MS/MS: liquid chromatography-tandem mass spectrometry
LGE: lateral ganglionic eminence
MAP2: microtubule associated protein 2
MRPs: mitochondrial ribosomal protein families
MS: mass spectrometry
MSN: medium spiny neurons
NGN2: Neurogenin 2
NSC: neural stem cells
PAX6: paired box 6
PCA: principal component analysis
POLDIP3: DNA polymerase delta interacting protein 3
SCA: spinocerebellar ataxia
SOX2: SRY-box transcription factor 2
SRSFs: serine and arginine rich splicing factor families
SVA: SINE-VNTR-Alu
TAF1: TATA-binding protein associated factor 1
TAF1–32i: TAF1 aberrant transcript that is composed of the canonical exon32 spliced to a cryptic exon in intron 32
TFIID: transcription factor II D
TFs: transcription factors
TUBB3: tubulin beta 3 class III
WGCNA: weighted coexpression network analysis
XDP: X-linked dystonia-parkinsonism
XIC: extracted ion chromatogram
YY1: YY1 transcription factor
