# Supplementary figures and images for: Proteomic analysis of X-linked dystonia parkinsonism disease striatal neurons reveals altered RNA metabolism and splicing
Source: Neurobiol Dis. Author manuscript; Available in PMC 2024 May 20. (PMC11103251; doi:10.1016/j.nbd.2023.106367)

## Slide 1
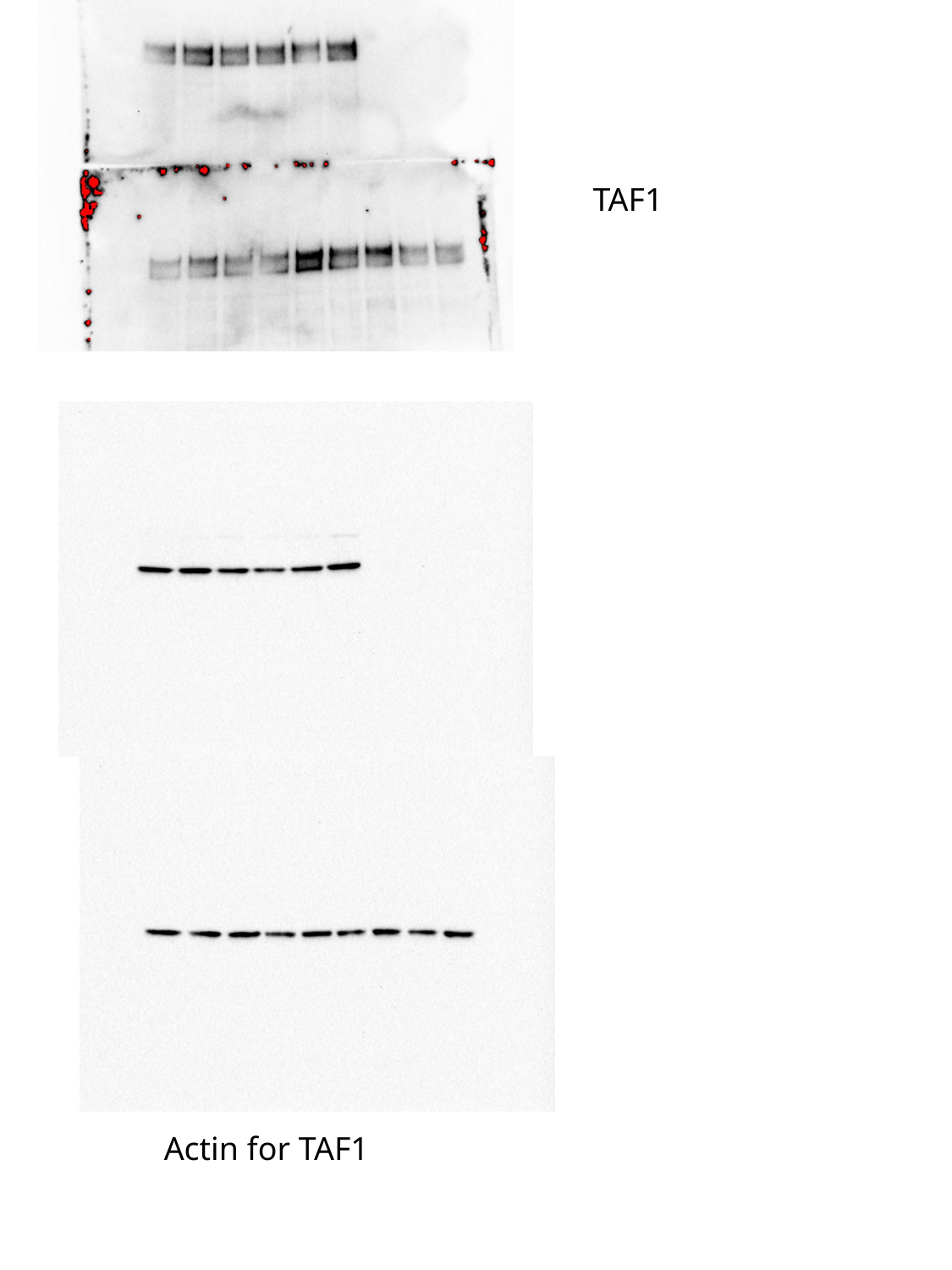

TAF1
Actin for TAF1

Supplement: suppl material western blots [file NIHMS1990109-supplement-suppl_material_western_blots.zip › mmc11.pptx]
