# Supplementary material for: Proteomic analysis of X-linked dystonia parkinsonism disease striatal neurons reveals altered RNA metabolism and splicing
Source: Neurobiol Dis. Author manuscript; Available in PMC 2024 May 20. (PMC11103251; doi:10.1016/j.nbd.2023.106367)

Supplementary Figure 1

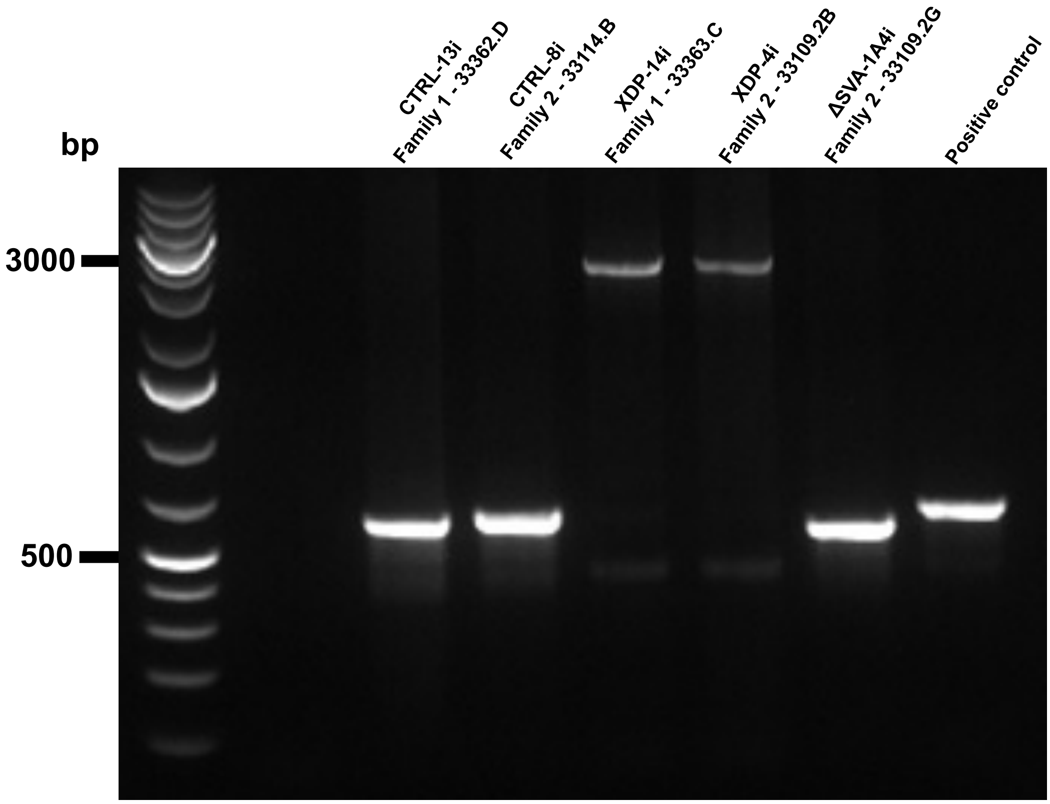

Supplementary Figure 2

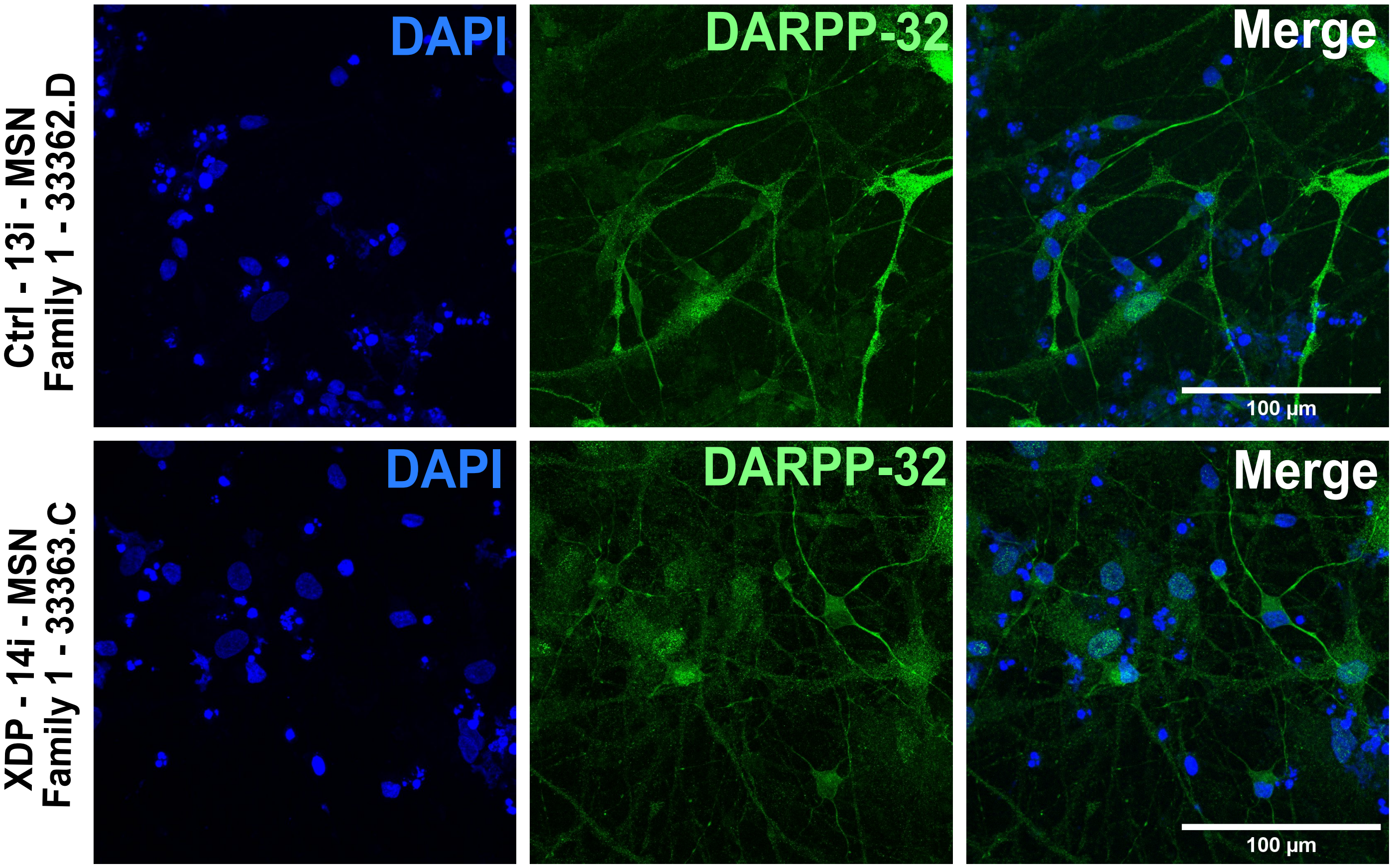

Supplementary Figure 3

A. Sample-specific hybrid DDA-DIA spectral libraries

Spectral library	# Precursors	# Peptides	# Proteins groups	# Proteins
**NSC**	81,872	58,855	6,089	12,218
**MSN**	57,651	43,335	5,092	10,531

B. Retention time calibration for one NSC replicate

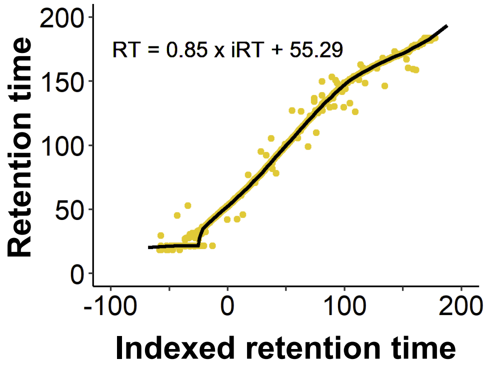

C. Distribution of CV in NSC dataset

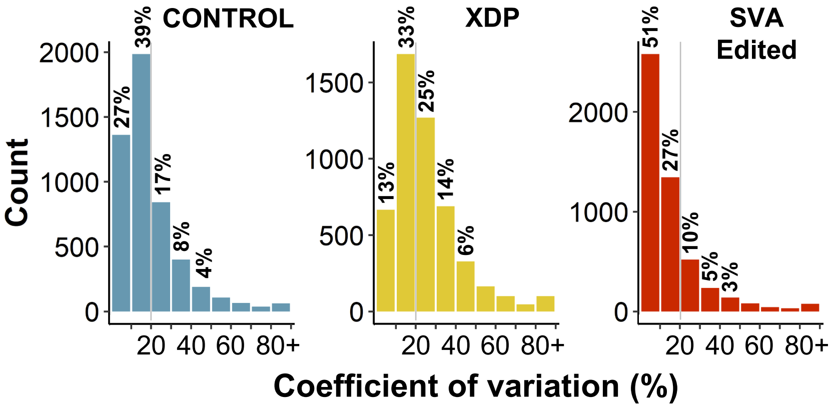

D. Retention time calibration for one MSN replicate

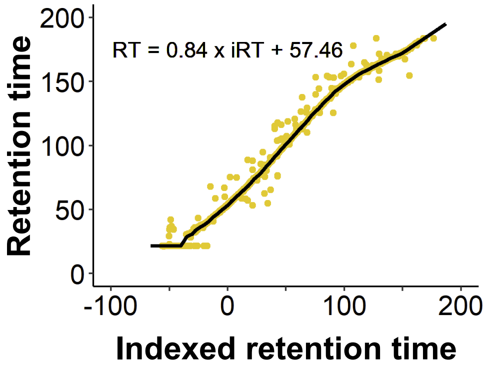

E. Distribution of CV in MSN dataset

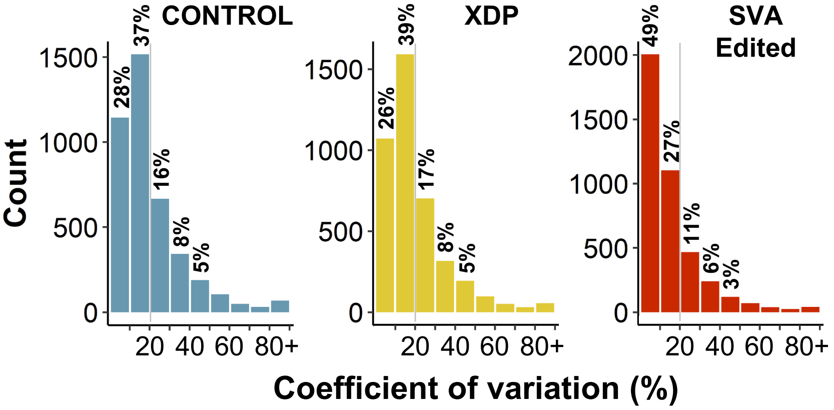

Supplementary Figure 4

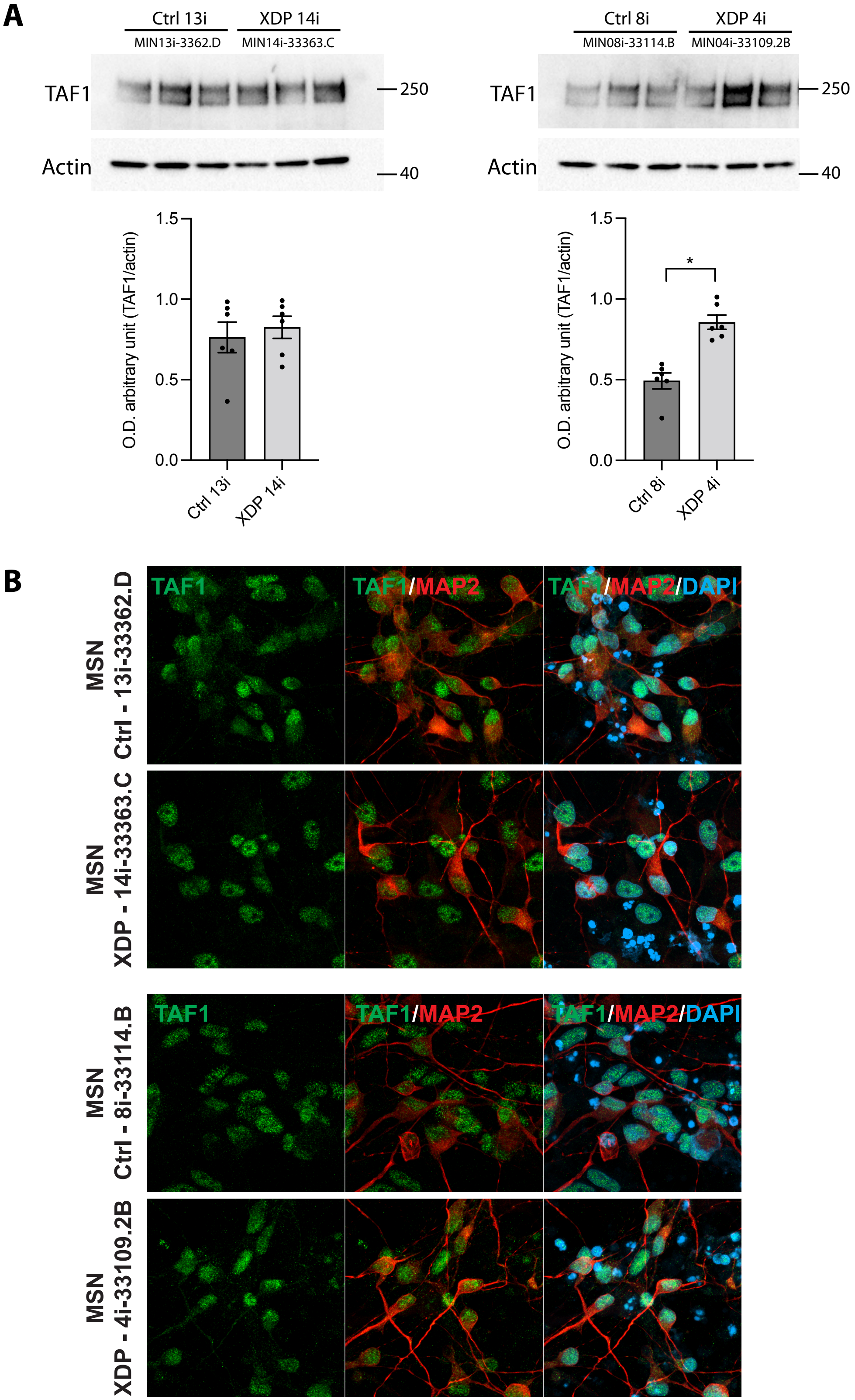

Supplementary Figure 5

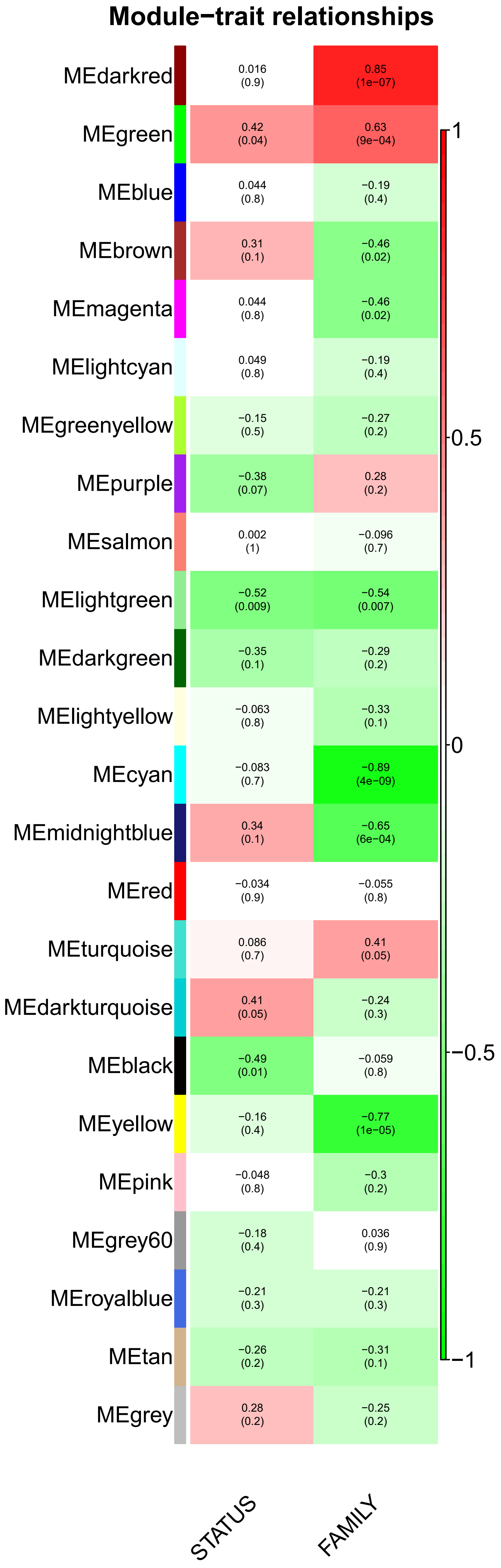

Supplementary Figure 6

Light green module

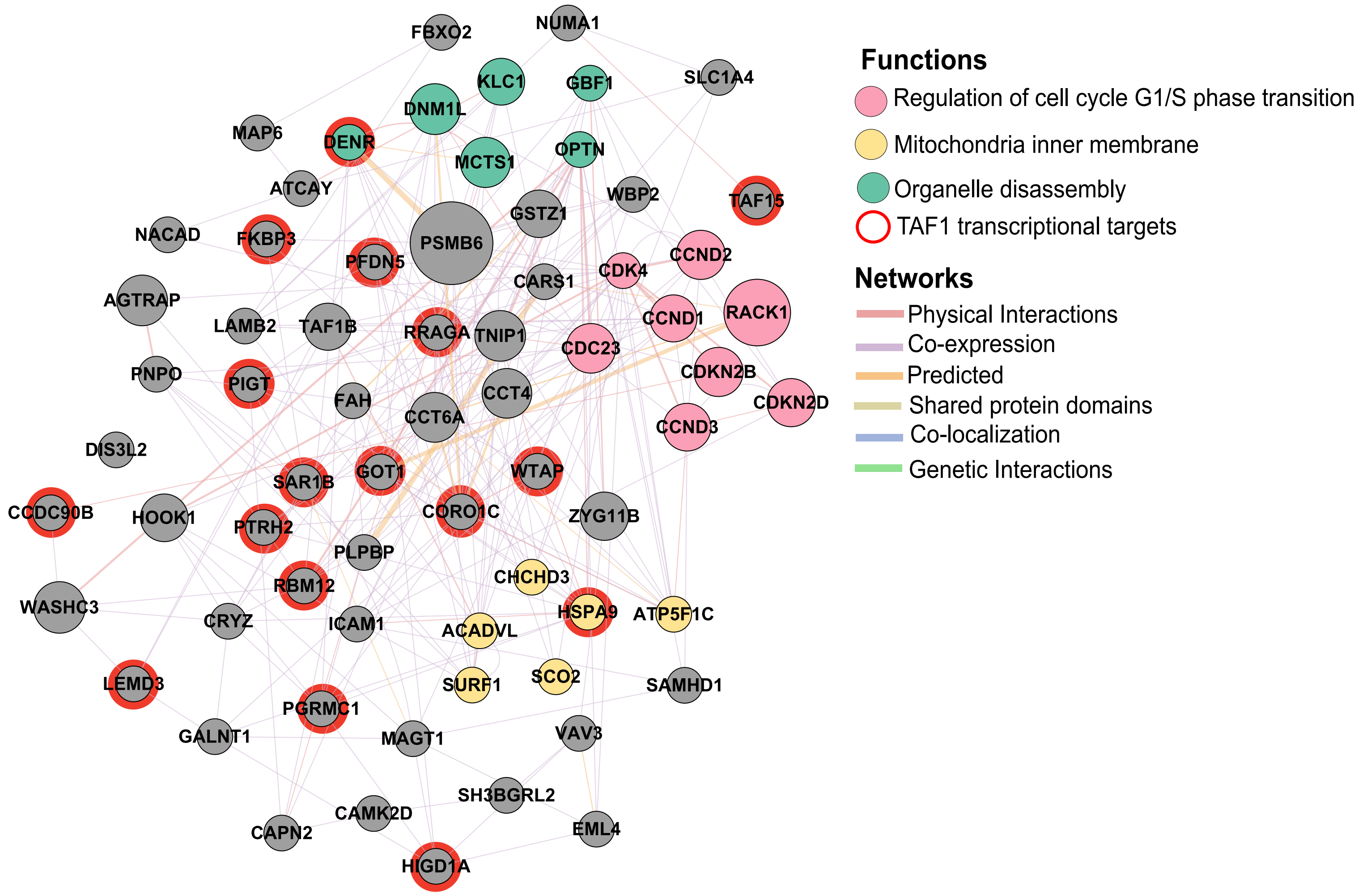

Supplement: Suppl Figures 1-6 [file NIHMS1990109-supplement-Suppl_Figures_1-6.pdf]
